# Expansion of eastern Mediterranean Middle Paleolithic into the desert region in early marine isotopic stage 5

**DOI:** 10.1038/s41598-022-08296-9

**Published:** 2022-03-16

**Authors:** Omry Barzilai, Maya Oron, Naomi Porat, Dustin White, Rhys Timms, Simon Blockley, André Zular, Yoav Avni, Galina Faershtein, Steve Weiner, Elisabetta Boaretto

**Affiliations:** 1 Archaeological Research Department, Israel Antiquities Authority, POB 586, 91004 Jerusalem, Israel; 2 Max Planck-Weizmann Center for Integrative Archaeology and Anthropology, DANGOOR Research Accelerator Mass Spectrometry Laboratory, Weizmann Institute of Science, 7610001 Rehovot, Israel; 3 Institute of Archaeology, The Hebrew University of Jerusalem, Mt. Scopus, 91905 Jerusalem, Israel; 4 Geological Survey of Israel, 32 Yesha’ayahu Leibowitz St., 9692100 Jerusalem, Israel; 5 Centre for Quaternary Research, Department of Geography, University of London, Egham, UK; 6 Department of Chemistry, University of York, York, UK; 7 Department of Earth and Planetary Sciences, Weizmann Institute of Science, 7610001 Rehovot, Israel

**Keywords:** Anthropology, Archaeology, Cultural evolution

## Abstract

Marine Isotopic Stage 5 is associated with wetter climatic conditions in the Saharo-Arabian deserts. This stage also corresponds to the establishment of Middle Paleolithic hominins and their associated material culture in two geographical provinces in southwest Asia—the Eastern Mediterranean woodland and the Arabian Peninsula desert. The lithic industry of the Eastern Mediterranean is characterized by the centripetal Levallois method, whereas the Nubian Levallois method characterizes the populations of the Arabian desert. The Negev Desert, situated between these regions is a key area to comprehend population movement in correlation to climatic zones. This investigation addresses the nature of the Middle Paleolithic settlement in the Negev Desert during MIS 5 by studying the site of Nahal Aqev. High resolution chronological results based on luminescence dating and cryptotephra show the site was occupied from MIS 5e to MIS 5d. The lithic industries at Nahal Aqev are dominated by centripetal Levallois core method. These data demonstrate that Nahal Aqev is much closer in its cultural attributes to the Eastern Mediterranean Middle Paleolithic than to the Arabian Desert entity. We conclude that Nahal Aqev represents an expansion of Middle Paleolithic groups from the Mediterranean woodland into the desert, triggered by better climatic conditions. These groups possibly interacted with hominin groups bearing the Nubian core tradition from the vast region of Arabia.

## Introduction

**Figure 1:**
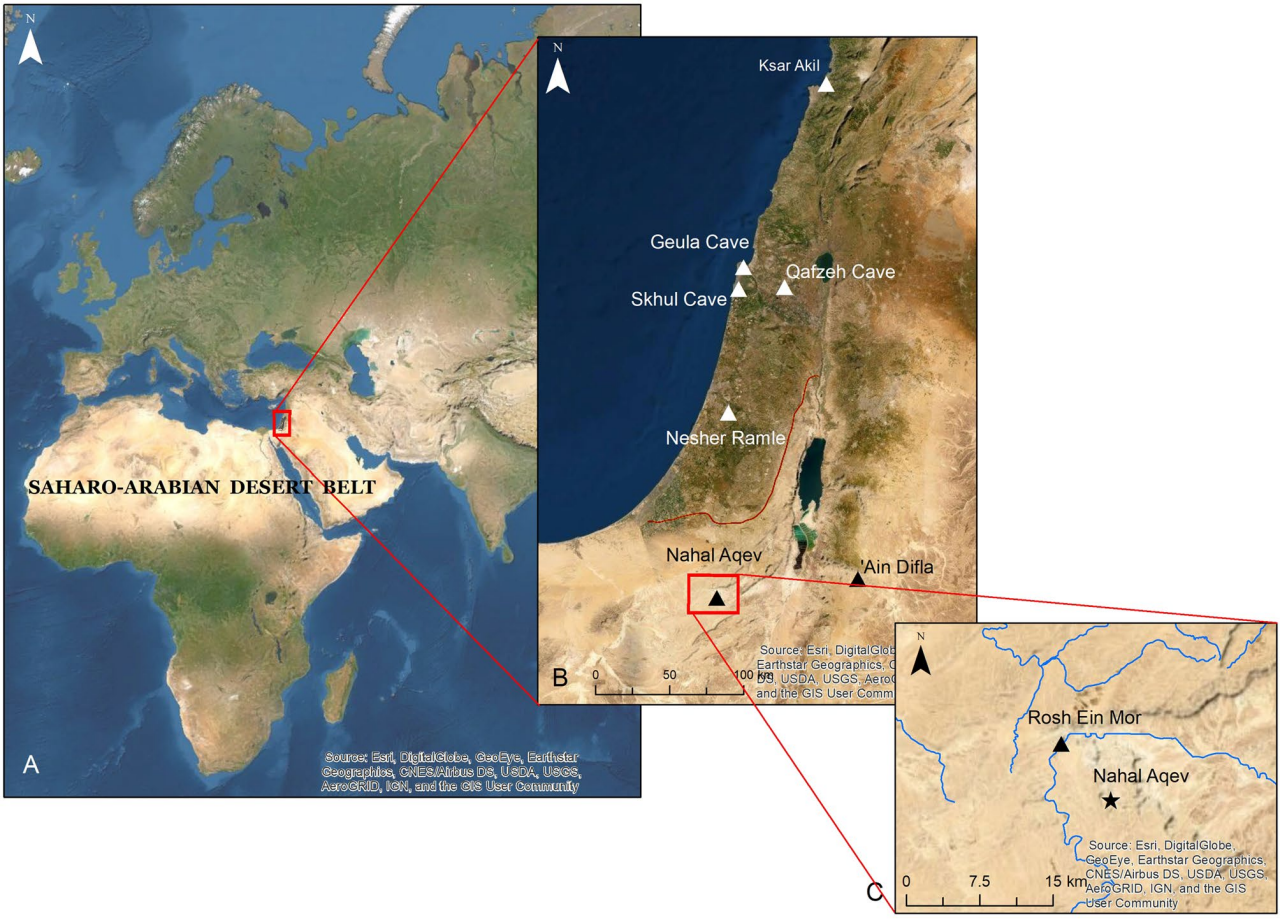
Location of Nahal Aqev and the Negev desert. (**A**) The Saharo-Arabian desert belt. (**B**) Middle Paleolithic sites dated to MIS 5 in the Levant. Note the boundary between the Mediterranean woodland and the Negev desert (orange line). (**C**) The location on Nahal Aqev within the central Negev. All rights reserved to IAA GIS-research lab. Base maps provided by ESRI online database. Figure 1 was prepared by M. Birkenfeld (Israel Antiquities Authority, IAA).

The eastern Saharo-Arabian desert is a major physical barrier between East Africa and Southwest Asia; two regions which played a key role in the evolution and spread of modern humans: (Fig. 1A)^1,2^. Multi-disciplinary studies carried out over the last two decades have shown that this arid belt was subjected to several wet episodes, which narrowed its geographical boundaries^3–7^. The strongest and most recognized episode, evident in various records, occurred in MIS 5 (130–80 kya), which is the timespan associated with hominin and faunal expansions to south Arabia and the Levantine corridor^8–14^. During this phase, the Arabian desert sustained lakes and wetlands which attracted hominins, most likely from African origins, bearing Nubian Levallois core technology^15–19^. Some of these populations are thought to have expanded further to the Thar Desert to the east, whereas others presumably diffused north to the Negev Desert^20,21^.

Marine Isotopic Stage (MIS) 5 hominins are well represented in the adjoining region to the north of Arabia, the Levant, and in particular the Mediterranean woodland region. Independent studies of several cave sites i.e., Skhul, Qafzeh and Geula show continuous Middle Paleolithic occupation during MIS 5^22–27^ (Fig. 1B). Other sites, such as the open-air site of Nesher Ramla, display earlier occupational continuity from MIS 6^28–30^. Notably, the hominins in these sites employed a centripetal Levallois reduction strategy, and occasionally used ochre pigments and produced symbolic artifacts and shell ornaments^31–35^.

The climatic reconstruction for the Mediterranean woodland region during the MIS 5 is based on speleothem studies from Soreq Cave, Israel, supported by the presence of specific faunal remains, clearly indicates an increase in warmer climate and possibly summer precipitation^8,13,36–38^. An additional study on speleothems from the Negev shows the desert region was also subjected to wetter climate during in MIS 5, but less pronounced^3^.

While the MIS 5 archaeological record in the Levantine Mediterranean woodland is well known, the Levantine deserts, including the Negev, are less well documented. Although the Negev has been surveyed by several expeditions, the number of Middle Paleolithic sites in the Negev is comparatively small and mostly confined to the central Negev^39–42^. The cultural affiliation and chronologies are insecure and debated^43–46^, and so are their paleoenvironmental settings. Accordingly, the Middle Paleolithic of the Negev desert cannot currently be correlated with neighboring regions of the Mediterranean woodland and south Arabia despite the evidence for wetter climatic conditions in these regions during MIS 5.

### The Negev Desert

The Negev Desert is a confined region between the Mediterranean Sea and the Red Sea at the northern part of the Saharo-Arabian desert belt (Fig. 1). This region has a rich record of well-preserved sedimentary units, some of which include relics of Middle Paleolithic sites^39,40,47–49^ (SI-1 Appendix). Most of the Middle Paleolithic occurrences are in the central Negev and are characterized by Levallois core technology^41^. Recent work in previously surveyed findspots also identified evidence for Nubian Levallois core tradition in the central and the southern Negev^21,49,50^.

The two preserved and most documented sites in the central Negev are Rosh Ein Mor and Nahal Aqev^51,52^ (Fig. 1C). Both were excavated in the 1970s and were ascribed to the early phase of the Middle Paleolithic (Tabun D tradition) based on characteristics of their lithic industries^53^. A later study proposed that this early phase of lithic tradition persisted in the Negev Desert to the later Middle Paleolithic^54^. The absolute chronology of these sites is undetermined as several dating attempts provided non-reproducible results for Rosh Ein Mor, or relied on dates from travertine contexts outside the Nahal Aqev site^43–46^.

Consequently, the Middle Paleolithic sequence of the central Negev is not well established, making it difficult to estimate its role in broader research topics such as population movements into the arid regions during wetter periods, or possible connections with neighboring regions. Hence, this study aims at resolving these issues based on the new excavations at the site of Nahal Aqev.

### The site of Nahal Aqev

**Figure 2:**
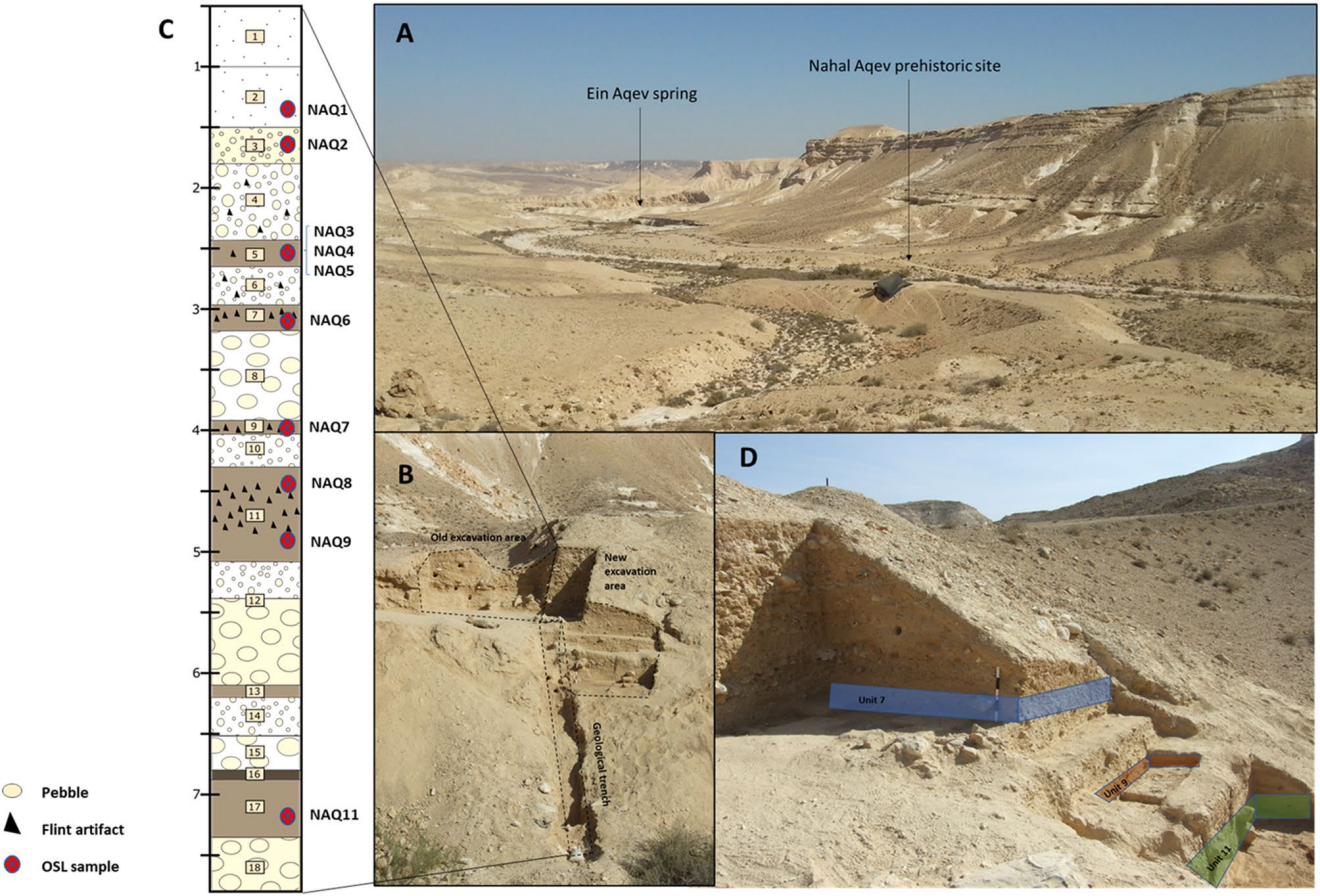
The Nahal Aqev study area. (**A**) Location of the site and the current Ein Aqev spring situated 500 m downstream (view to north). The excavation area is under the black shade. (**B**) Old and new excavation areas and the geological trench (view to east). (**C**) Compiled stratigraphic section of Nahal Aqev. Scale bar (left) in meters. (**D**) Southern view of the geological units bearing archaeological horizons, color-shaded for clarity (Unit 7, light blue; Unit 9, orange; Unit 11, light green). Figure 2 was prepared by O. Barzilai and M. Oron.

Nahal Aqev was initially excavated in the 1970s^52^. The site is embedded within an alluvial terrace deposited near the bottom of the upper step of Aqev canyon at an elevation of 430 m asl (Fig. 2A). In 2015–2016 we conducted two excavation seasons at Nahal Aqev to determine its stratigraphy and absolute chronology^55^. The fieldwork was initially carried out adjacent to the old excavation area of 1974 (Fig. 2B). The sections of the old excavation were cleaned, and a geological trench was dug from the excavation area down to the base of the terrace along its western slope. In addition, a new area comprising 12 m^2^ was opened to the south (SI-2 Appendix), in order to expose and excavate new archaeological layers discovered in the trench and sections. Eighteen sedimentological units were identified in the new excavations (Fig. 2C). All units present a similar mineralogical composition consisting mainly of calcite, clays and quartz, and small amounts of feldspars, heavy minerals and gypsum.

The upper two Units 1‒2, are composed of eolian sediments accumulated on the terrace surface probably after its abandonment as the active fluvial system ceased to exist (SI-1 Appendix). Units 3‒18 are composed of fluvial and colluvial coarse to fine-grained clasts forming discrete units alternating between fine-grained silty sands with some small pebbles (Units 3, 5, 7, 11, and 17), thin units of clayish fine-grain gray sediment (Units 9, 13, and 16), coarse gravels (Units 10 and 14) and high energy colluvial units with some bigger pebbles and large stones (Units 4, 6, 8, 12, 15, and 18). Flint artifacts were found in most of the units, but mainly in the upper part of the section (Units 1**‒**11). In Units 7, 9, and 11 the flint artifacts are in primary deposition, whereas in the other Units they seem to be in secondary deposition, as the artifacts were randomly dispersed with no horizontal order. Also, many of these artifacts display fractures and abraded lateral edges which indicate that they were subjected to post-depositional movement.

**Figure 3:**
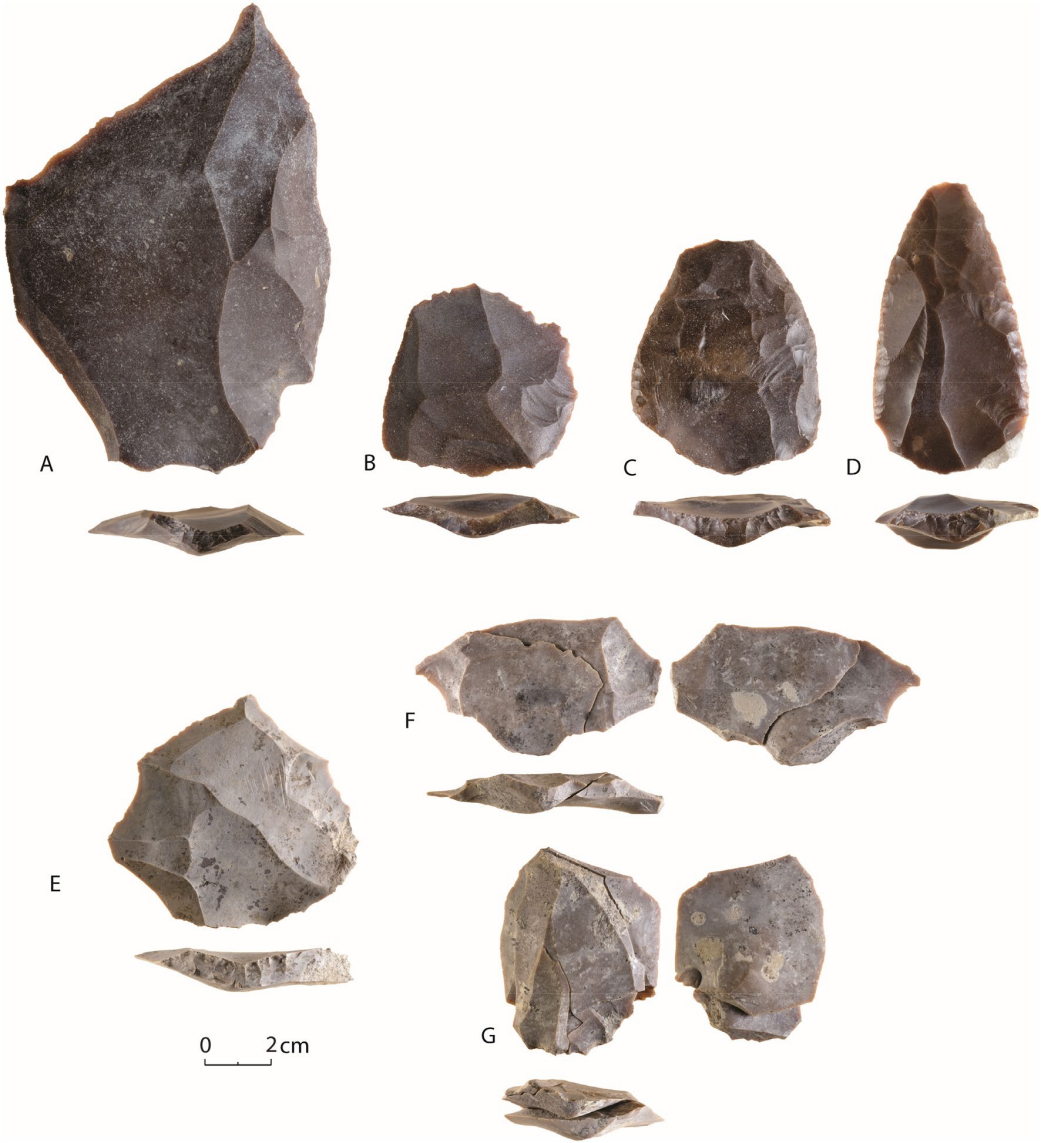
Characteristic lithic artifacts produced by Centripetal Levallois core technology from Nahal Aqev Unit 11 (**A**–**E**) and Unit 7 (**F**, **G**). (**A**) Large Levallois flake with bidirectional scars. (**B**) Levallois flake with a centripetal scar pattern. (**C**, **D**). Scrapers on Levallois flakes. (**E**) Levallois flake with a centripetal scar pattern. (**F**, **G**) Refitted aggregates of Levallois flakes and by products. Figure 3 was prepared by D. Gazit and C. Herch (IAA).

The new excavations revealed three preserved archaeological horizons in Units 7, 9, and 11—all displaying concentrations of horizontally embedded artifacts in fine silt sediments (Fig. 2D). The lithic artifacts in these assemblages are extremely fresh, and refitting is evident within the assemblage of Unit 7 (Fig. 3). The lowermost archaeological horizon, and hence the earliest occupation at the site, is embedded within Unit 11. The horizon is composed of silty-sand sediments formed between two gravel units. The archaeological horizon is about 30 cm thick, but the main concentration of finds is in its lower 15 cm. This level is rich and very well preserved, with horizontally embedded flint artifacts (N = 4877) along with a few fragments of ostrich eggshell (SII Appendix, Fig. S2.5). Concentrations of burnt flints and ash sediment indicate the presence of a hearth feature. Patches of gypsum within the ashy sediment imply the firewood included Tamarix species (SI-3 Appendix).

The lithic assemblage from Unit 11 is dominated by a centripetal Levallois method which seems like the only core technology used (Fig. 3A–E). The presence of a few large Levallois blanks with bidirectional scar patterns, suggest the centripetal reduction sequence probably began as bidirectional and in due course of the knapping converted to circumferential. Centripetal Levallois method is also dominant in the upper Units 9 and 7, and in the surface material (Fig. 3F, G) (SI-4 Appendix). Nevertheless, more technological variability is apparent in the upper horizons as expressed by the presence of unidirectional convergent Levallois flaking as well as non-Levallois flaking methods for flakes and bladelet production (mainly single platform cores and cores on flakes).

### Luminescence dating (OSL) chronology

Ten samples were collected from the entire section (Table 1; Fig. 2C). Details of sampling in the field, sample preparation and measurements, dose rate evaluations and age calculations are presented in SI-5 Appendix. Quartz and alkali feldspar (KF) in the size range of 90–125 µm were extracted and purified using routine laboratory procedures^56^.

**Table 1:** Field and laboratory data for samples collected for luminescence dating.

Lab code	Description	Depth (m)	Aliquots/grains	Dose rate (Gy/ka)	OD (%)	De (Gy)	Age (ka)
NAQ-1	Lower unit 2—fluvial with small stones within silty matrix and almost no artifacts	1.2		1.82 ± 0.09			
MG			23/25		39	77 ± 5	42 ± 3
SG			79/80		76	78 ± 7	42 ± 4
NAQ-2	Unit 3- fine grained silt with some flint artifacts in low frequencies, probably layer 2 of Marks	1.55		1.57 ± 0.08			
MG			22/25		35	138 ± 7	88 ± 6
SG			99/100		82	101 ± 9	67 ± 7
NAQ-3	Upper Unit 5—fine grained silt with flint artifacts, probably level 3 of Marks, above the main flint concentration	1.95					
MG			22/25	1.71 ± 0.05	58	132 ± 10	77 ± 6
SG			73/73		62	107 ± 8	63 ± 5
KF			6/6	2.21 ± 0.09	6	188 ± 5	85 ± 4
KF*							96 ± 9
NAQ-4	Lower Unit 5 under main flint concentration	2.3					
MG			25/25	1.47 ± 0.07	30	144 ± 9	98 ± 8
SG			136/137		43	127 ± 5	86 ± 6
KF			6/6	1.95 ± 0.10	6	207 ± 6	107 ± 6
KF*							124 ± 8
NAQ-6	Lower unit 5, fine grained silty unit with flint artifacts	2.4					
MG			24/25	1.84 ± 0.05	34	121 ± 8	66 ± 5
SG			120/120		53	136 ± 7	74 ± 5
KF			6/6	2.36 ± 0.12	6	199 ± 6	84 ± 5
KF*							92 ± 6
NAQ-5	Unit 7- silty with a clear archaeological horizon, within archaeological layer	3.0					
MG			25/25	1.48 ± 0.06	40	145 ± 12	98 ± 9
SG			146/147		44	137 ± 6	92 ± 6
KF			6/6	1.96 ± 0.10	0	222 ± 3	114 ± 6
KF*							131 ± 23
NAQ-7	Unit 9, clayish gray sediment	3.7					
MG			25/25	1.39 ± 0.05	30	134 ± 9	97 ± 7
SG			56/62		50	190 ± 11	137 ± 9
KF			6/6	1.86 ± 0.09	5	216 ± 5	116 ± 6
KF*							132 ± 7
NAQ-8	Unit 11, silty sediment a clear archaeological	4.5					
MG			25/25	1.70 ± 0.08	37	140 ± 11	83 ± 8
SG			69/70		50	139 ± 9	82 ± 7
KF			6/6	2.17 ± 0.11	4	225 ± 5	103 ± 6
KF*							117 ± 7
NAQ-9	Base of Unit 11	5.0					
MG			23/25	1.58 ± 0.07	45	167 ± 12	106 ± 9
SG			176/177		56	148 ± 7	94 ± 6
KF			6/6	2.04 ± 0.10	0	261 ± 4	118 ± 6
KF*							134 ± 7
NAQ-11	Unit 17, silty sediment	5.4					
MG			22/25	1.21 ± 0.061	37	125 ± 7	104 ± 8
SG			62/64		63	138 ± 11	115 ± 11

Samples arranged by burial depth. MG, multi-grain quartz; SG, micro-aliquot quartz; KF, multi-grain alkali feldspar. KF* are KF ages corrected for fading. Aliquots/grains is the number of aliquots or grains used for De calculations out of those accepted. OD—Overdispersion, the scatter within the sample beyond that expected from instrumental noise. Average and errors on the De values were calculated using the central age model^58^. Ages are in thousands of years. For details of dose rates and other parameters used for age calculations, see SI.

**Figure 4:**
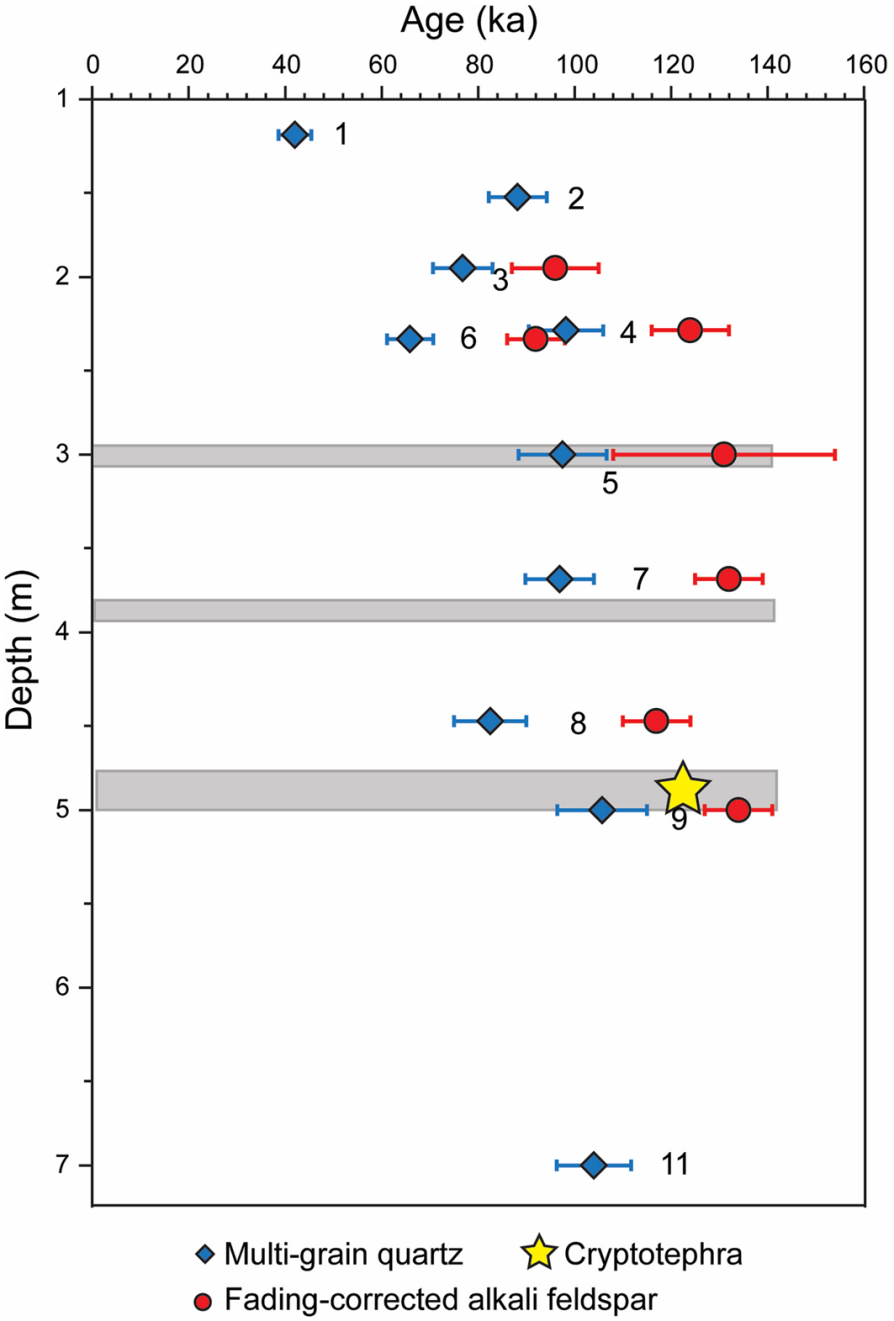
Luminescence ages as a function of depth in the sediment profile of Nahal Aqev. Horizontal bands show the archaeological layers 7, 9 and 11 (from top to bottom) and the star shows the level and estimated age of the cryptotephra (see below). Figure 4 was prepared by N. Porat.

Table 1 and Fig. 4 present the luminescence ages for quartz and KF multi-grains. Single grain De values have a roughly normal distribution and do not show evidence of partial bleaching (SI Appendix, Fig. S5.2 h). KF ages were corrected for fading as in Huntley and Lamoth^57^; this increases the KF ages by ~ 10–15%, and the errors on the ages. The ages range from ~ 40 to ~ 135 ka for the top and base of the section, respectively. For all samples, the natural OSL and pIR-IR_250_ signals are bright and decay fast (SI Appendix, Fig. S5.2). The dose distributions are mostly normal (SI Appendix, Fig S5.2), although MG OSL measurement have medium over-dispersion (OD) values (Table 1).

The pIR-IR_250_ KF ages are mostly older than the quartz ages and the difference generally increases with depth (Table 1). This probably indicates that for the older samples, the quartz De values are nearing saturation and their ages are thus somewhat underestimated. Indeed, SI Appendix, Fig. S5.5 shows that while the De values of the quartz hardly increase below 2 m, those of the KF continue to increase with depth. Thus, we consider the pIR-IR_250_ ages on KF to be more reliable.

The ages increase with depth up to ~ 2 m, and below that are almost constant (Fig. 4), possibly indicating rapid sedimentation. Exceptional is sample NAQ-6. It is from the same layer as NAQ-4 but from different sections of the excavation walls (SI Appendix, Fig. S5.3) and is somewhat younger than the overlying NAQ-3, whereas NAQ-4 follows the stratigraphic order. It is worth noting that the De values of samples NAQ-4 and NAQ-6 are similar for both quartz and KF (SI Appendix, Fig. S5.5), suggesting that perhaps the dose rates of either sample were not estimated correctly.

Using the pIR-IR_250_ KF fading-corrected ages, our best estimates for the ages of archaeological Units 7, 9 and 11 are as follows: Sample NAQ-5 gives an age of 131 ± 23 ka for Unit 7; sample NAQ-7 gives an age of 132 ± 7 ka for Unit 9; and samples NAQ-8 and NAQ-9 bracket Unit 11 to between 117 ± 7 ka and 134 ± 7 ka. Overall, the ages of the archaeological horizons fall within MIS 5e (Fig. 4).

### Cryptotephra

**Figure 5:**
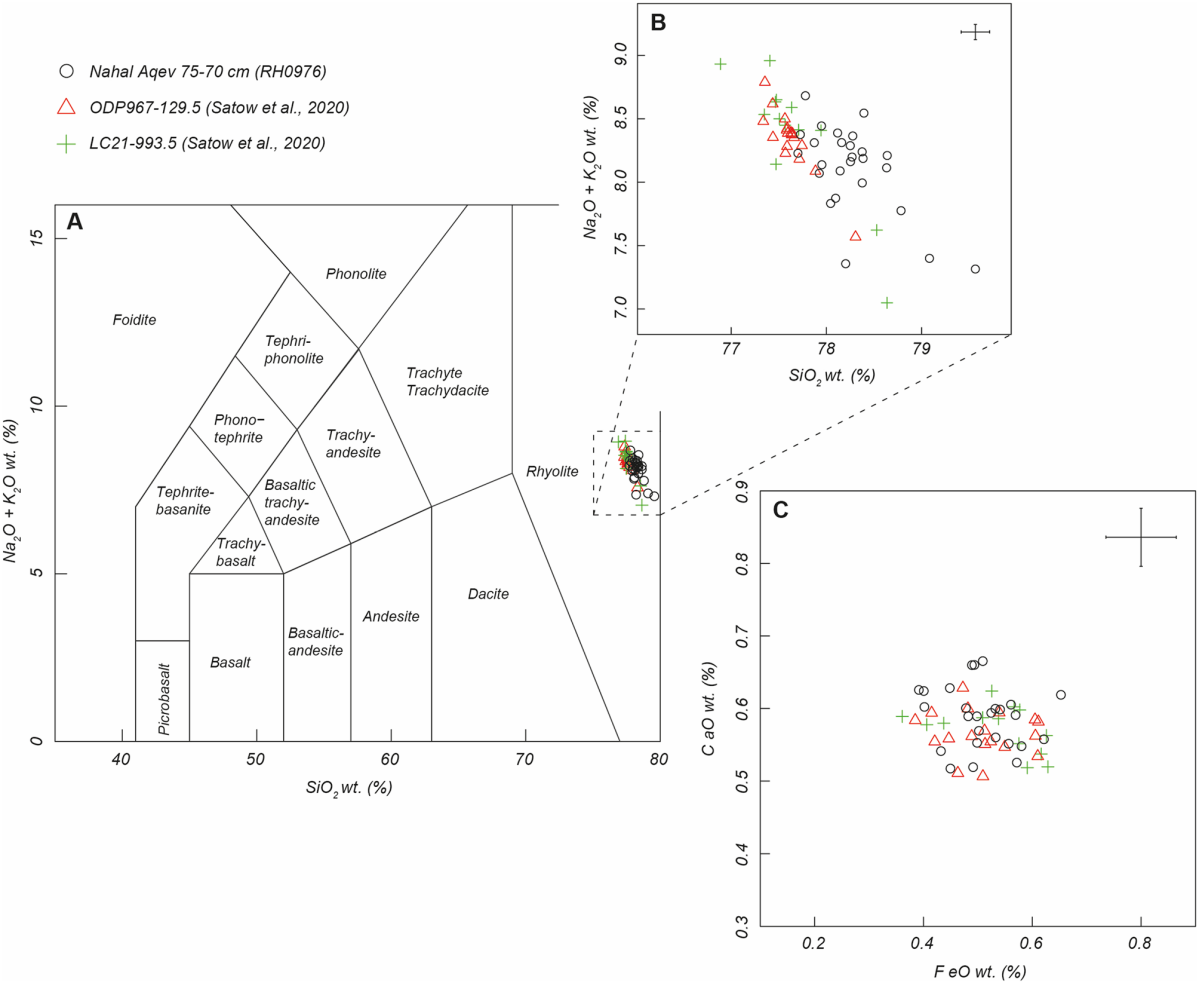
Geochemical composition of the Nahal Aqev tephra and correlation to MIS 5e, Sapropel S5 tephra from ODP-967 and LC-21 (values normalized on water free basis). (**A**) Total Alkali Silica (TAS) classification based on Le Bas et al.^64^, (**B**) inset of TAS plot, (**C**) FeO vs CaO wt. (%). Error bars represent 2 SD of replicate analyses of Lipari glass standards (Table S6.2). Additional geochemical plots demonstrating a clear correlation on all Oxides is available in SI Appendix, Fig. S6.1. Figure 5 was prepared by R. Timms and S. Blockley.

Samples for cryptotephra analysis were collected from the new excavation at 5 cm consecutive and contiguous intervals from 2 sampling columns (SI-6 Appendix). Column 1 spanning Unit 3 to Unit 7 and Columns 2/2A extending from Unit 9 to Unit 11. In the laboratory, volcanic glass shard (cryptotephra) extraction and quantification followed published guidelines^59,60^. A distinct peak in glass shard concentrations was identified in Unit 11 and within the archaeological horizon (SI Appendix, Fig. S6.1). Chemical characterization of this ‘peak’ was conducted at the WDS-EPMA facility at the University of Edinburgh following Hayward^61^. Major and minor elements indicate the glass shards are of a calc-alkaline rhyolite composition (Fig. 5). Chemical comparison to regionally significant volcanic centers and sedimentary archives containing cryptotephra, suggests that the Nahal Aqev tephra correlates to a highly evolved eruption from the Anatolian Acıgöl system or the Hellenic volcanoes, specifically Kos. This is due to the high SiO_2_ and very low CaO and FeO wt% values which are uncommon in tephra found across the eastern Mediterranean^62^. Within the broad time window provided by the luminescence dating on the site there is only one known eruption with these chemical characteristics. A tephra with an identical match to the chemistry from Nahal Aqev has been traced as a cryptotephra in early MIS5e sediments in the marine cores ODP-967 and LC-21^61^. The tephra is located in early Sapropel S5 deposits in both cores, and in LC21, which is located close to Santorini, the tephra is the lowest of multiple horizons in this period, although only the lowermost tephra is stratigraphically secure and has a chemical match to the Nahal Aqev tephra. In core ODP-967, which is the easternmost of the two records, located to the south of Cyprus, the tephra is the only one detected in S5 deposits and is at a much higher shard concentration. This fits with the high shard concentrations in Nahal Aqev (SI-6 Appendix) and there is a clear chemical match between the tephra (Fig. 5) (SI Appendix, Fig. S6.1). The tephra in ODP-967 and LC-21 is a unique stratigraphic marker for MIS5e and is dated to 126.4 ± 2^63^.

## Discussion

This study of Nahal Aqev revises our understanding of the chronology of the Middle Paleolithic and its lithic traditions in the central Negev. The luminescence ages together with the cryptotephra provide an exceptionally trustworthy chronology for Nahal Aqev which dates the Middle Paleolithic occupations at the site from MIS 5e to MIS 5d (Fig. 4). Considering the age discrepancies at Rosh Ein Mor, these dates currently set Nahal Aqev as the earliest and only dated Middle Paleolithic site in the central Negev. Nahal Aqev can now be correlated to the adjoining regions.

The nature of the occupations at Nahal Aqev is of a base camp, with domestic activities such as flint knapping and use of fire taking place. Fragments of ostrich eggshells, found within the archaeological horizon of Unit 11, could represent food wastes, or broken liquid containers. Notable is the hearth feature in this horizon that utilized Tamarix as fuel wood. The presence of ostrich eggshells and Tamarix at Nahal Aqev imply the broad environmental conditions in the central Negev did not change completely Mediterranean during MIS 5 as it maintained arid/semi-arid species. This may correspond to the limited deposition of speleothems between 126 and 118 ka in the Ashalim, Mizpe Ramon and Ma'aleh HaMeishar caves^3^.

The increased precipitation in the Negev during MIS 5 is thought to have originated from the Mediterranean Sea^3^. The identification of the Nahal Aqev crypotephra source with volcanoes from the Eastern Mediterranean region supports this affiliation. This precipitation must have augmented groundwater sources in the Negev, as attested in the local speleothems, which in turn, enhanced the flow of the local springs as evident by the number of fossilized springs and by extensive travertine surfaces within the immediate surroundings of the site, as well as in the southern Negev^43,65^. Accordingly, it is reasonable to assume that the paleo Ein Aqev spring was available all year around and supported fauna and flora, providing sufficient food resources to sustain hominin groups in the central Negev.

**Figure 6:**
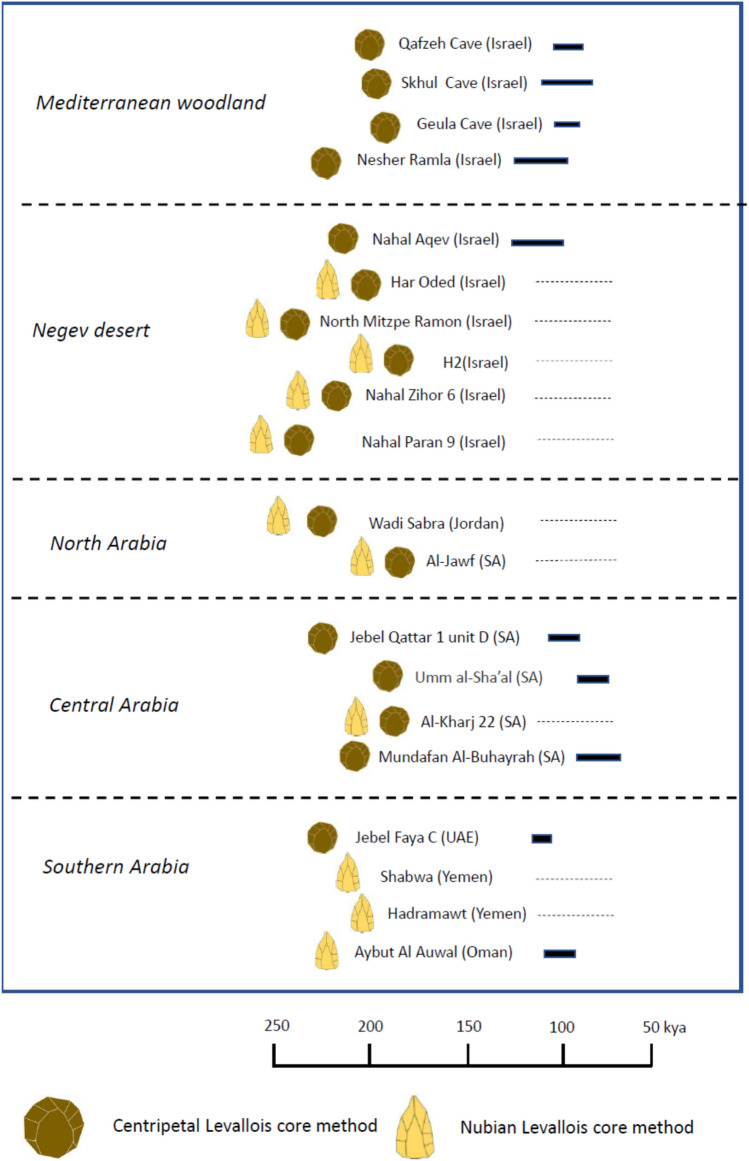
Range of ages (bars on the right) and lithic industry of MIS 5 sites from the Mediterranean woodland, Negev desert and the Arabian desert^9,15–17,24,27,28,66,68–72^. Note that Centripetal Levallois and Nubian Levallois dominate the Mediterranean woodland and southern Arabia, respectively. Solid bars stand for numerical ages and dashed line for age estimates. The only dated site in the Negev desert (this study), Nahal Aqev, is dominated by Centripetal Levallois. Figure 6 was prepared by O. Barzilai.

The lithic assemblages from the three archaeological occupations at Nahal Aqev are characterized by centripetal Levallois core technology. An assessment of contemporary MIS 5 lithic assemblages from the Mediterranean woodland and Arabia regions attests two distinguishable lithic traditions (Fig. 6). The first is the centripetal Levallois system, corresponding to the Richter et al. "Middle East contextual area"^66^, which was the main mode of production at sites from the Mediterranean woodland region, e.g. Qafzeh Cave, Skhul Cave, Geula Cave and Nesher Ramla^26–30,67^. The other lithic tradition is the Nubian Levallois core technology whose distribution corresponds strongly with southern Arabia^15–18^.

Nahal Aqev, although located in a desert region, is overwhelmingly dominated by centripetal Levallois core technology and as such this site is ascribed to the MIS 5 Mediterranean cultural group. Remarkably, the arid regions of the Negev and Arabia do not bear MIS 6 sites or assemblages, suggesting the arid region was hardly inhabited at this stage. This is in contrast to the Mediterranean woodland, which was populated (though less than in MIS 5). This population used the centripetal Levallois method^27–29^. Accordingly, we propose that the centripetal Levallois method expanded from the Mediterranean core area to the arid regions of the Negev and Arabia during MIS 5.

The Mediterranean woodland and southern Arabia are separated by a vast currently hyper arid region extending over a distance of ca. 2000 km. During MIS 5 this region had favorable hotspots that included lakes and permanent springs^14,19^ which could have sustained hominin groups traveling between the two regions. Various field expeditions revealed dozens of find spots and surface sites in the Negev and Arabia bearing elements from both lithic traditions: the centripetal and the Nubian^15–18,21,41,49,50,68–72^ (Fig. 6). Accordingly, it is reasonable to assume these MIS 5 occurrences in this buffer zone were associated and/or connected in one way or another to the two core areas. Consequently, it is interpreted that the area between the Mediterranean woodland and south Arabia was the interaction zone between these two lithic traditions who were "on the move" to the greening deserts from opposite directions. Such a scenario corresponds with the interaction zone or exchange of ideas between two "contextual areas" as suggested also by Rose and Marks^72^ and Richter et al.^66^.

While the Arabian region is conceived to incorporate new populations during MIS 5, the Mediterranean woodland province shows evidence for cultural continuity between MIS 6 and MIS 5 populations^11,29^. Much was written on the implications for the appearance of the Nubian Levallois core technology in Arabia during MIS 5. One accepted explanation is that this technology reflects migration of African populations into the desert^9,11^. These populations are thought to have moved further to the east to India, and possibly also to the fringes of the Levant to the north^20,21^.

The presence of indistinguishable lithic technology in the Negev desert and the Mediterranean woodland in time periods corresponding to better climatic conditions is interpreted as expansion of the Mediterranean traditions into the desert (e.g. the Aurignacian techno-complex during MIS 3)^73,74^. Accordingly, we suggest that the dominance of the centripetal Levallois tradition at Nahal Aqev reflects an expansion of Middle Paleolithic hominin groups into the desert region from the Mediterranean woodland following better climatic conditions.

All in all, during MIS 5 we witness the expansion of two lithic traditions into the arid lands; the centripetal Levallois from the Mediterranean woodland at the north part of the desert belt, and the Nubian Levallois from Africa to south Arabia.

## Conclusions

Nahal Aqev is currently the earliest most securely dated Middle Paleolithic site in the central Negev. The oldest occupation of the site, based on luminescence and cryptotephra, corresponds to MIS 5e, when improved climatic conditions prevailed in the Saharo-Arabian deserts.

The dominance of centripetal Levallois method at Nahal Aqev ties the site to contemporary Middle Paleolithic sites bearing a similar lithic tradition in the Mediterranean woodland region. The emergence of this technology in the Negev during MIS 5e most likely reflects a southern expansion of Middle Paleolithic groups from the Mediterranean woodland due to better climatic conditions in the desert.

## Materials and methods

### Luminescence

The equivalent dose (De) values were measured using single aliquot regenerative dose (SAR) protocols^75^ (Appendix Table S2); on TL/OSL Risø readers equipped with calibrated beta sources. For quartz, De was measured on 1-mm aliquots (multi-grain; MG) with ~ 50 grains per aliquot. Single grains (SG) of quartz were measured to check for partial bleaching, using standard SG discs with an array of 10 × 10 holes, each with a diameter and depth of 300 µm. The latter are effectively micro-aliquots, as hole diameter is 300 µm whereas grain size is 90–125 µm, such that in each grain hole there were 3–4 grains^76^. Twenty-five multi-grain aliquots and 400–500 grain holes were measured for each sample. Micro-aliquot data were screened for further data processing using criteria defined in Porat et al.^77^.

For KF, De was measured on 1-mm aliquots (~ 50 grains) on stainless steel cups using the SAR protocol^78^. The post-infrared (pIR-IR) signal measured at 250 °C (pIR-IR_250_) was selected after this temperature was found to balance between ease of bleaching of the signal and relatively low anomalous fading in samples with a similar geological source^56^. Anomalous fading was measured on four samples for up to 50 h following Buylaert et al.^79^, averaging 1.3 ± 0.2% per decade.

Average De values were calculated using all measurements and the central age model, which assumes that the grains are distributed around a central value^58^. Dose rates were calculated from the concentrations of the radioactive elements U, Th and K, measured by Inductively coupled plasma (ICP) mass spectrometry (MS) or ICP optical emission spectrometry (OES). Cosmic dose rates were evaluated from current burial depth and time averaged moisture content was estimated at 5% to 8%, depending on current burial depths. For dose rate details see SI Appendix, Table S5.1.

### Cryptotephra

Individual sediment sub-samples weighing ~ 2–3 g were extracted from the 41 bulk sediment samples, with three to four of these combined to form composite or ‘scan’ samples. 13 scan samples were produced to span the sampled Nahal Aqev sequence.

Scan samples were placed in an oven overnight at 105 °C to establish their dry weight and combusted in a laboratory muffle furnace for two hours at 550 °C to remove organic detritus. Residual material was immersed in 10% Hydrochloric acid (HCl) to dissolve carbonates before being passed through nylon sieve meshes with apertures of 125 and 15 μm. All material in this size fraction was retained for further processing.

The extraction of volcanic glass shards was conducted using a stepped density separation procedure and the inert heavy liquid sodium polytungstate (SPT) (**Na6 (H2W12O40) H2O**)^59^. An initial ‘cleaning’ phase was conducted using the SPT at a specific gravity of 2.00 g/cm3, and an ‘extraction’ phase using SPT with a specific gravity of 2.55 g/cm^3^ (51). Material from the extraction phase was pipetted onto glass microscope slides and mounted under a coverslip using Canada balsam.

Microscope slides were examined using an Olympus CX-41 transmitted light microscope. Slides were traversed systematically, and counts were conducted at 20 × magnification, an objective with 40 × magnification was used to examine morphological detail and to assist in distinguishing volcanic glass shards from non-glass detrital ‘mimics’.

## Supplementary Information

Supplementary Information.
