# Supplementary material for: Expansion of eastern Mediterranean Middle Paleolithic into the desert region in early marine isotopic stage 5
Source: Sci Rep. 2022 Mar 16;12:4466. doi: 10.1038/s41598-022-08296-9 (PMC8927120; doi:10.1038/s41598-022-08296-9)
Supplement: Supplementary file 1 — Supplementary Information. [file 41598_2022_8296_MOESM1_ESM.pdf]

Supplementary Information

Expansion of Eastern Mediterranean Middle Paleolithic into the Desert Region in early Marine Isotopic Stage 5

Omry Barzilai, Maya Oron, Naomi Porat, Dustin White, Rhys Timms, Simon Blockley, André Zular, Yoav Avni, Galina Faershtein, Steve Weiner, Elisabetta Boaretto

Section 1. Geology of the region and the formation of the Nahal Aqev terrace system

Nahal Aqev is a tributary of Nahal Zin, which is one of the largest drainage basins in the Dead Sea region, occupying more than 1,000 km^2 (1). The lower part of Nahal (stream) Aqev is composed of a deep canyon with two permanent springs - Ein Aqev Upper and Ein Aqev Lower. The canyon, ca. 200 m deep, displays two steps of incision corresponding to erosional stages which shaped the regional drainage system (Fig. S1.1).

The upper step, ca. 3-4 km wide valley, was formed in the first erosional stage. The bottom of this valley is composed of the chalky Horsha Formation, overtopped by massive limestone layers of the Matred Formation. The lower step, formed in the second erosional stage, comprises a narrow ravine, 100-400 m wide (Fig. S1.1A). The lower step cuts through chalk and flint layers of the Mor Formation, overlain by the massive chalky-limestone layers of the Nizzana Formation. The configuration of the two steps of the Nahal Aqev canyon marks two distinct cycles in the landscape evolution of the central Negev region. Following the general uplift of the Negev highlands in the Middle Miocene ca. 16-14 Ma (2), the embryonic Aqev stream began to incise within the Avedat Plateau, forming the upper valley. At this stage the Aqev stream drained the Avedat plateau toward the Mediterranean Sea through Nahal Besor (3). During the Pliocene and Early Pleistocene, ca. 3-2 Ma ago, conglomerates of the Ahuzam Formation accumulated at the bottom of this relatively wide valley, ca. 100 m above its present level.

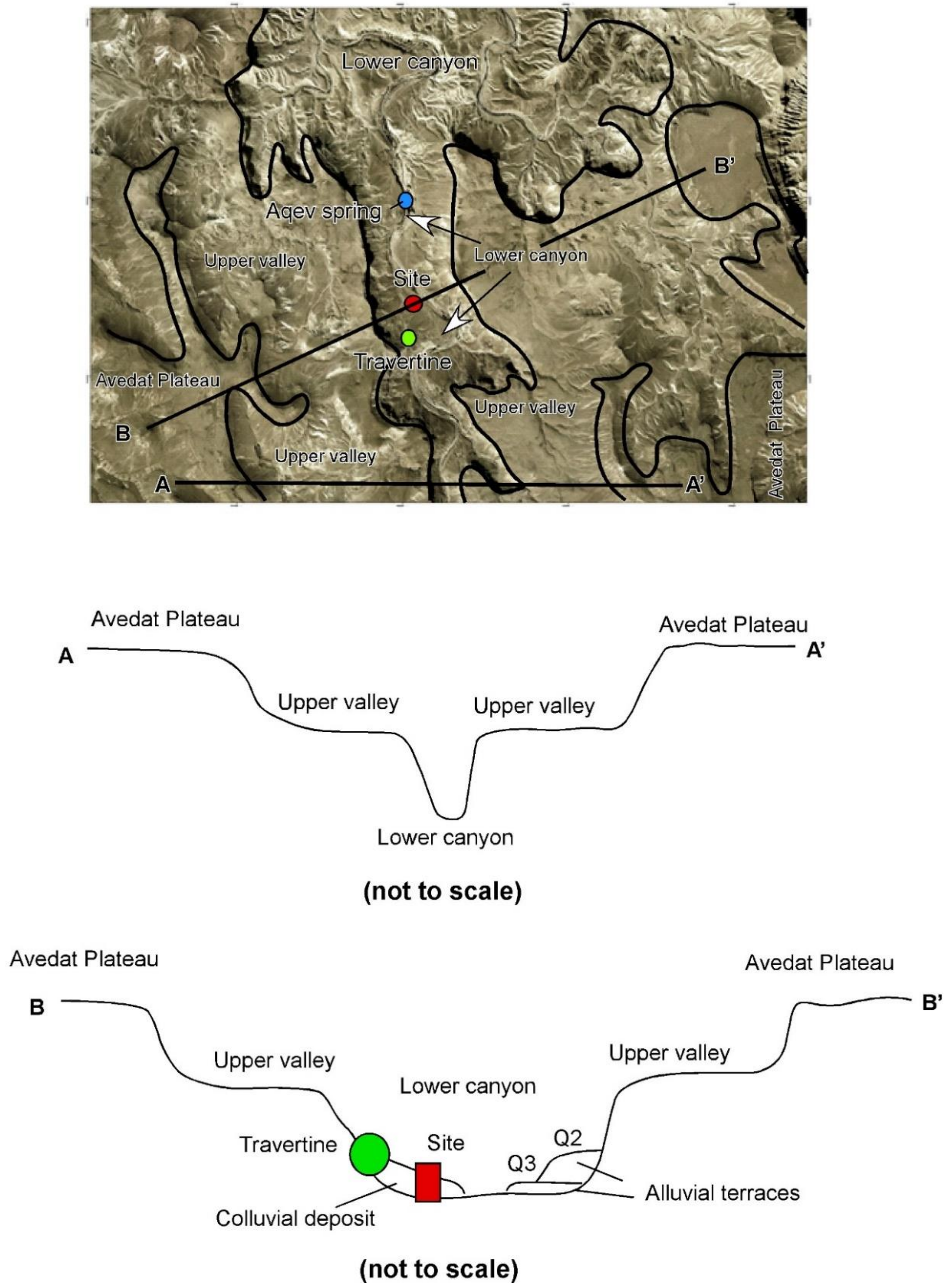

Fig. S1.1: Orthophoto and cross sections (A-A', B-B') of Nahal Aqev showing site location, terraces and travertines.

The second stage of incision is related to a widespread tectonic phase that uplifted, tilted and faulted the entire Negev toward the Arava Valley in the east, at ca. 1.5-1 Ma (3-4). This eastward tilt caused a general shift of the drainage orientation in Nahal Zin and its tributaries, including Nahal Aqev, toward the Dead Sea basin (3). This process was accompanied by a deep incision of Nahal Aqev, forming its present level, ca. 100 m below its previous Early Pleistocene level.

Alluvial terraces in the lower Aqev canyon

The incision of the lower canyon was a gradual process that took place during the Pleistocene. Nevertheless, during this time, two phases of deposition of fluvial and alluvial deposits occurred (Fig. S1.1B). These depositions formed terraces labeled Q2 and Q3, following the definition of Quaternary morphostratigraphic units presented in Avni and Wieler (5) and Avni et al., (6-7).

The Q2 terrace system is associated with the gradual aggradation of alluvial deposits accumulating to an elevation of ca. 30-20 m above the present stream bed along the mainstream channel and its major tributaries. At present Q2 terraces are preserved in several patches in Nahal Aqev and associated with local travertines. The terraces are composed of a mixture of rock fragments, gravels and desert dust and are correlated with talus and colluvium deposits that accumulated simultaneously along the steep slopes of the lower canyon (Fig. S1.2). The Q2 terrace system and its associated talus deposits are dated roughly to 200-100 ka (6-7). However, more research is needed for more precise dating of this phase of accumulation.

After the end of the depositional phase of the Q2 terrace, it was affected by an erosional phase corresponding with MIS 5 that incised some of the Q2 terraces down to the bedrock. This phase is associated with regional travertines that were deposited directly on the exposed bedrock or on top of preserved Q2 talus relicts. In some cases, local accumulation of colluvium deposits is recognized, similar to the colluvium on which the archaeological site is embedded (Fig. S1.2).

The termination of MIS 5 was subsequently followed by a new accumulation phase of alluvial deposits which accumulated within the lower Nahal Aqev canyon, up to 8-10 m above the present streambed. These sediments formed a prominent fluvial terrace, marked as the Q3 terrace, that is mostly composed of desert dust, known also as desert loess (8), interbedded with rock fragments of local origin. The Q3 terraces are linked to the nearby canyon slopes and cliffs by colluvium mantels. In several locations in the Negev Highlands these terraces were dated to ca. 80-18 ka BP (7, 9-10).

Following the major Late Pleistocene–Holocene climate shift, dated to 14-12 Ka (10), Nahal Aqev began incising into the Q3 alluvial terraces to form its present stream bed that is slightly incised into the bedrock exposed below the base of the Q3 terrace.

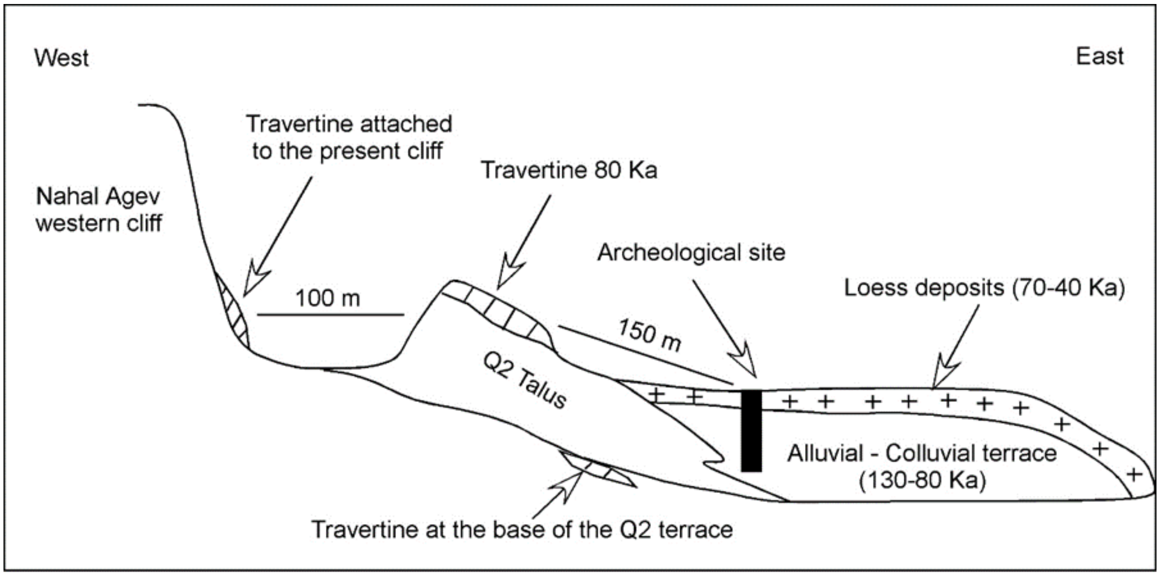

Fig. S1.2: Schematic section of Nahal Aqev and its immediate geomorphic units and surfaces.

The geomorphological setting of the archeological site

The archeological site is interbedded within a colluvium body that is composed of boulders, rock fragments and pebble units interbedded with fine-grained sediments, indicating high to low energy alternations during deposition. This colluvial – fluvial body developed downslope of the Q2 talus, forming a flat hill 100-150 m east of the main Nahal Aqev (Fig. S1.2). As indicated by its stratigraphic relations, this colluvial body developed after the accumulation of the Q2 talus and before the accumulation of the Q3 terraces along the main Nahal Aqev valley, probably during MIS 5.

The sediments composing the colluvial body were contributed from the small tributary with a catchment area of 187,500 m^2 that drains a sector of the upper valley of Nahal Aqev (Fig. S1.1) toward the main Nahal Aqev channel. After dropping through the near vertical cliff forming the western edge of the lower Aqev canyon, the drop in transportation energy caused the deposition of this colluvial body, originally shaped as a broad, low inclined fan, interbedded with the archeological site, at a distance of 250 m from the present cliff.

Travertines

Several travertines were found within the Aqev valley in the vicinity of the archeological site.

1. A massive travertine deposit, 80-100 cm thick, situated 150 m southwest of the archeological site (Fig. S1.3:A). This travertine accumulated on top of a talus, a remnant of Q2 level. Schwarcz et al. (11) dated this travertine to at least two intervals of accumulation - an older phase at ca. 200 ka, and a younger phase at 80 ka. The age of the older phase corresponds with the deposition time of the Q2 terraces and colluvial deposits that were mapped downstream of Nahal Aqev (5). The younger travertine deposit contains embedded MP artifacts (Fig. S1.3:B) which led Schwarcz et al. to propose that the spring and the site were contemporaneous (11).

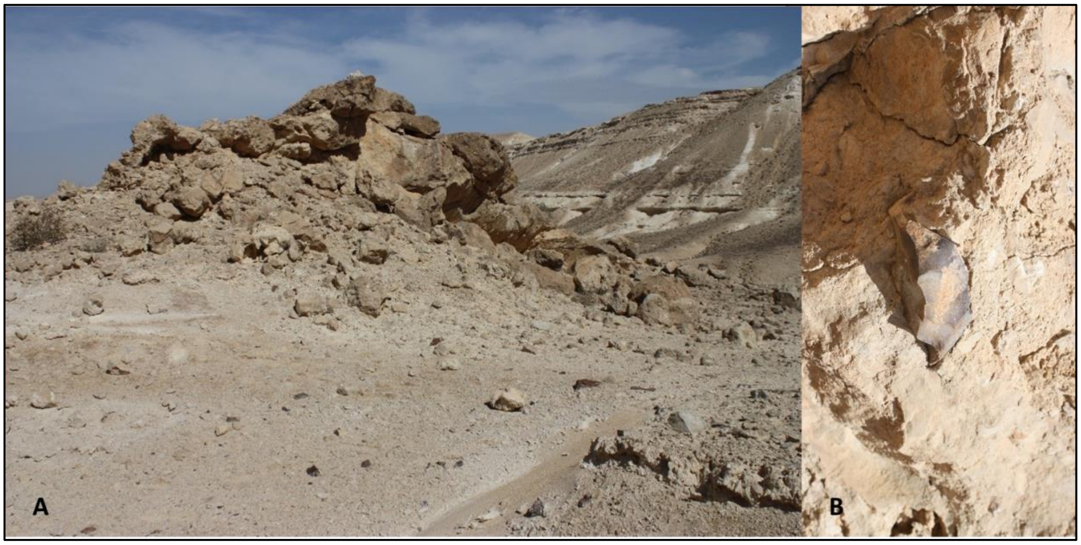

Figure S1.3. A. The southwest travertine; B. A Levallois core embedded in the travertine deposit.

2. A stream bed travertine, 15-20 cm thick, is located 50 m upstream of the archeological site at the contact between the chalk and flint beds of the Mor Formation and the alluvial deposit at the base of relict Q2 terraces (Fig. S1.2). The travertine is composed of small gravels cemented by calcium carbonate, resulting from carbonate-saturated spring-water that flowed along this contact before the deposition of Q2 terrace. Similar accumulations are visible along the main streambed of Nahal Aqev above the lower Aqev spring. At the moment no direct date is available for this travertine and its relation to the archeological site is unclear.

3. Holocene travertines were noted attached to the current cliffs in the vicinity of the site, 250 m to the southwest, which are 80-100 m above channel bed. At this location, a currently dry waterfall formed. The lower part of the waterfall is partly covered by a porous travertine, 20-50

cm thick, vertically attached to the cliff. This travertine indicates that in the past, a spring emerged from fissures in the rocks exposed by the waterfall, contributing water to the lower part of the tributary that flowed at the foot of the archeological site. The association of the travertine to the present cliff hints to its relatively young, probably Holocene, age.
At present travertines are accumulating near active springs and on wet rock surfaces in the vicinity to the Ein Aqev lower spring, some 500 m to the north of the archeological site.

Section 2. The site of Nahal Aqev

Investigations of the Middle Paleolithic (MP) period were conducted in the Negev during the 1970's in the framework of the Central Negev Project directed by Marks (12-14). Fieldwork performed in various areas in the Negev highland revealed a density of MP sites in the Avdat-Aqev region. Among the discovered sites were two larger occupations interpreted as basecamps, Rosh Ein Mor (D-15) and Nahal Aqev (D-35) sites, and nine ephemeral occurrences (15-18). Rosh Ein Mor and Nahal Aqev were the better-preserved stratified sites, each composed of several sedimentological units. The lithic industries of the two sites were assigned to the Early MP, as they were assumed to correspond to Tabun D-type of the Mediterranean woodland region (19). However, dating efforts of the two sites did not provide a clear assignment to the ascribed Early MP. At Rosh Ein Mor, uranium series dating of ostrich eggshells provided an age of ca. 200 ka, but thermoluminescence (TL) dating of burnt flints from the same contexts provided a range of 48-14 ka (20-21). Most recent dating of calcite crusts coating lithic artifacts have assigned Rosh Ein Mor to the Late MP corresponding to MIS 4 (22). Nahal Aqev was not directly dated, and its ascribed age of ca. 80 ka was based on uranium series dating of travertine deposits located ca. 150 meters southwest of the site (11).
The Nahal Aqev site is located at an elevation of 430 m asl, 500 m upstream from Ein Aqev spring (Fig. S2.1). Two excavation seasons were conducted at the site, in 1972 and 1974. In the first season two geological trenches were dug into the terrace deposits (Fig. S2.2). Trench 1 transected the terrace perpendicular to its long axis, from the east to the west. Trench 2 was dug along the western slope of the terrace. In the second season an excavation area was opened on the western side of the terrace, at the contact between Trench 1 and Trench 2. The excavations revealed three archaeological levels bearing flint artifacts from the MP period. Level 3, up to 70 cm thick, was the best preserved and had the thickest accumulation of lithic artifacts.

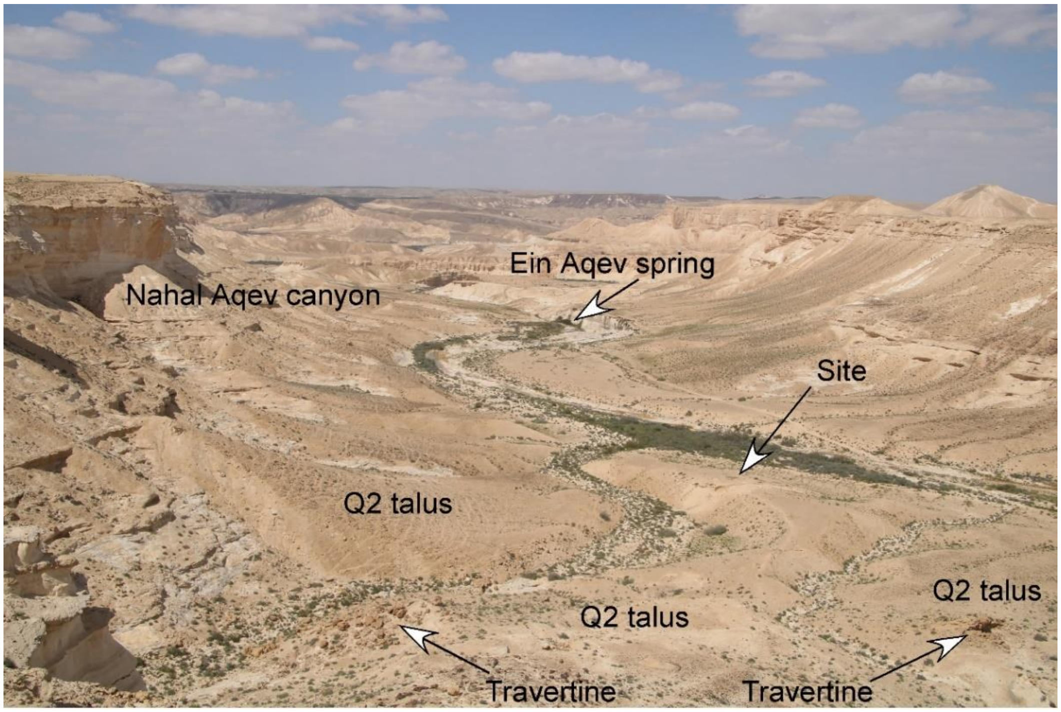

Figure S2.1. Nahal Aqev lower canyon - view to the north. Note that the site is located on the remains of a terrace talus, 500 m south of the lower Ein Aqev spring.

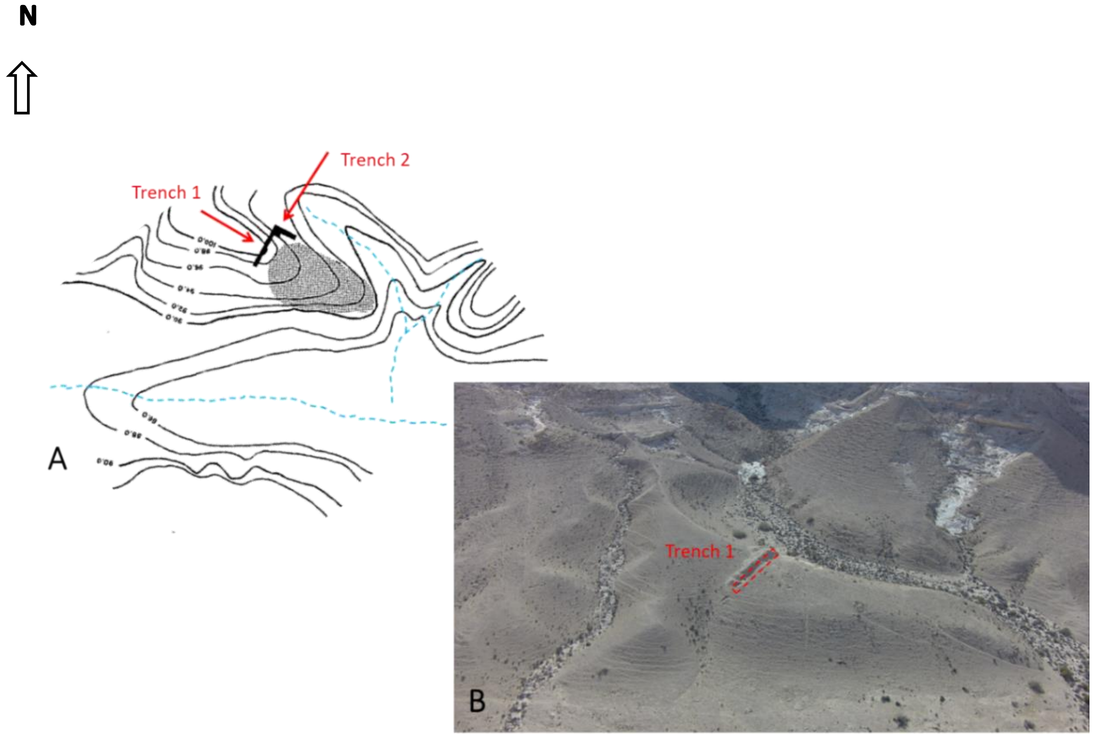

Figure S2.2. Nahal Aqev. A. Schematic plan modified after Munday (17). The two geological trenches are marked by red arrows. B. Aerial photo of Nahal Aqev taken in autumn 2015 (view to west). Note geological Trench 1 is still visible in the center of the terrace.

In 2015-2016 we conducted two excavation seasons at Nahal Aqev (permits # G-27/15; G-88/16 from the Israel Antiquities Authority) (23). The fieldwork was initially carried out in the old excavation area of 1974 (Fig. S2.3). The old excavation sections were cleaned, and a new area composed of 12 m² was opened to the south. In addition, a new geological trench (Trench 3, ca. 8 m long, 40 cm wide and 50 cm deep) was excavated from the new excavation area down to the base of the alluvial terrace along its western slope. In total, 18 sedimentological units representing one geomorphological sequence, were recognized (Fig. 2 manuscript). Units 1-7 occur in the excavation area and Units 8-18 in the geological trench. Excavation of archaeological layers was carried out in Units 3-7, 9 and 11 while the rest of the units were sectioned and cleaned but not excavated. Excavation was conducted in a 1X1 m grid in 10–5 cm spits. All sediments were dry sieved using 2 mm mesh.

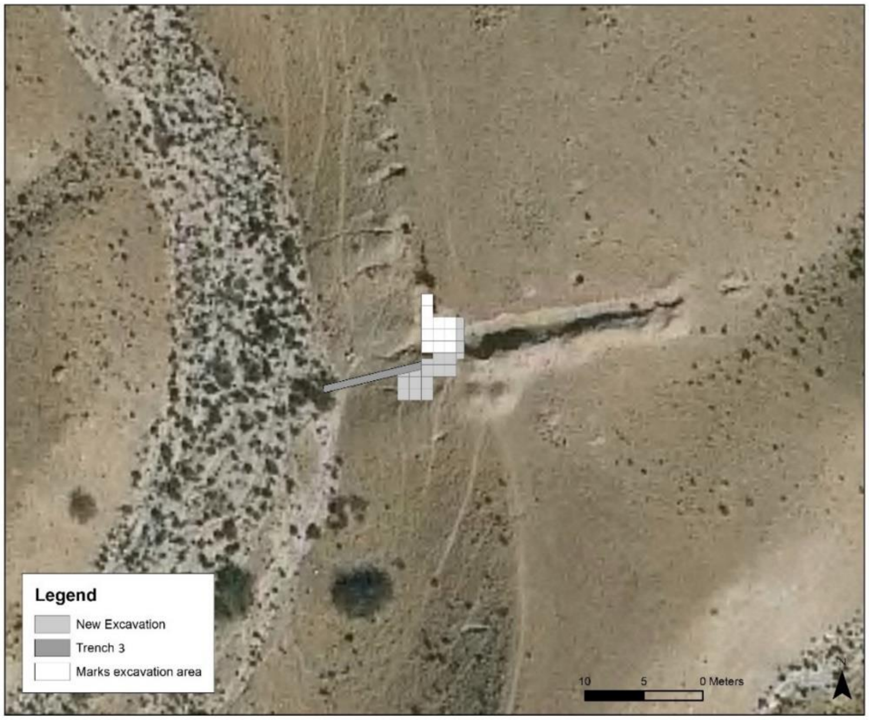

Figure S2.3. The site of Nahal Aqev (aerial view). Old excavation (north), new area (south), new geological trench (west).

Correlating the stratigraphy of the new excavation with the old one was not easy since no section drawings or photos of the excavation area were available. Still, based on the description of the stratigraphy of geological trench 1 by Goldberg (24; table 2-14) we were able to correlate the upper sedimentological units of the new excavation as shown in Figure S2.4.

The stratigraphy of the old excavation is composed of 10 lithological units (Figure S2.4 B). Within this sequence, three archaeological levels were observed: Level 1, corresponding to Goldberg's lithological unit 4 (Unit 3 in the new excavation). Level 2 deposited in lithological unit 8 (Unit 4 in the new excavation). And Level 3 that is described as a dense archaeological level, ca. 70 cm thick ascribed to lithological unit 10 at the base of the sequence (Units 5-6 in the new excavation).

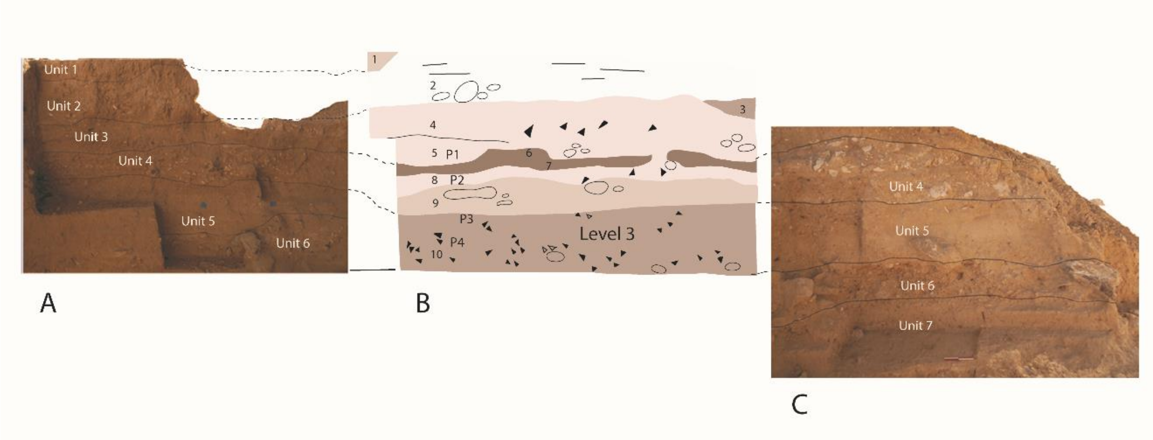

Figure S2.4 -Correlation between the old and the new excavations. A. East section 2015-2016 excavation. B. South profile of geological trench 1 (redrawn after Goldberg 1977: fig 2-8). Numbers 1-10 are Goldberg's lithological units. P1-4 point to the location of palynological samples. Black triangles represent lithic artifacts. C. South section 2015-2016 excavation. Note Level 3 from the old excavation (B) corresponds to Units 5 and 6 of the new excavation (A, C).

Archaeological horizons

Three archaeological horizons displaying concentrations of horizontally embedded artifacts were recognized in the new excavation (Fig. 2D manuscript). The uppermost archaeological horizon (Level A), embedded in Unit 7, was exposed in the old excavation area, below what was previously defined by Munday (17) as Level 3. This 20 cm thick level is composed of flint artifacts embedded horizontally within fine-grained silty sediment (Fig. S2.5). The level was excavated in an area of ~ 7 m^2, mainly below the old excavation area, but also in the western slope of the terrace.

Unit 9 is a thin layer composed of grey clayish sediment embedded between two bedded gravel units. The archaeological horizon, Level B, embedded in this unit was only exposed in a small area of ~ 1 m^2 and therfore our understanding of its nature is limited.

The lowermost archaeological horizon, Level C, and hence the earliest occupation at the site, is embedded in Unit 11 which is a silty sand sediment (Fig. S2.6). This horizon is ca. 30 cm thick, it is rich and very well preserved and is composed of flint artifact laying horizontally, ostrich eggshell fragments and a hearth.

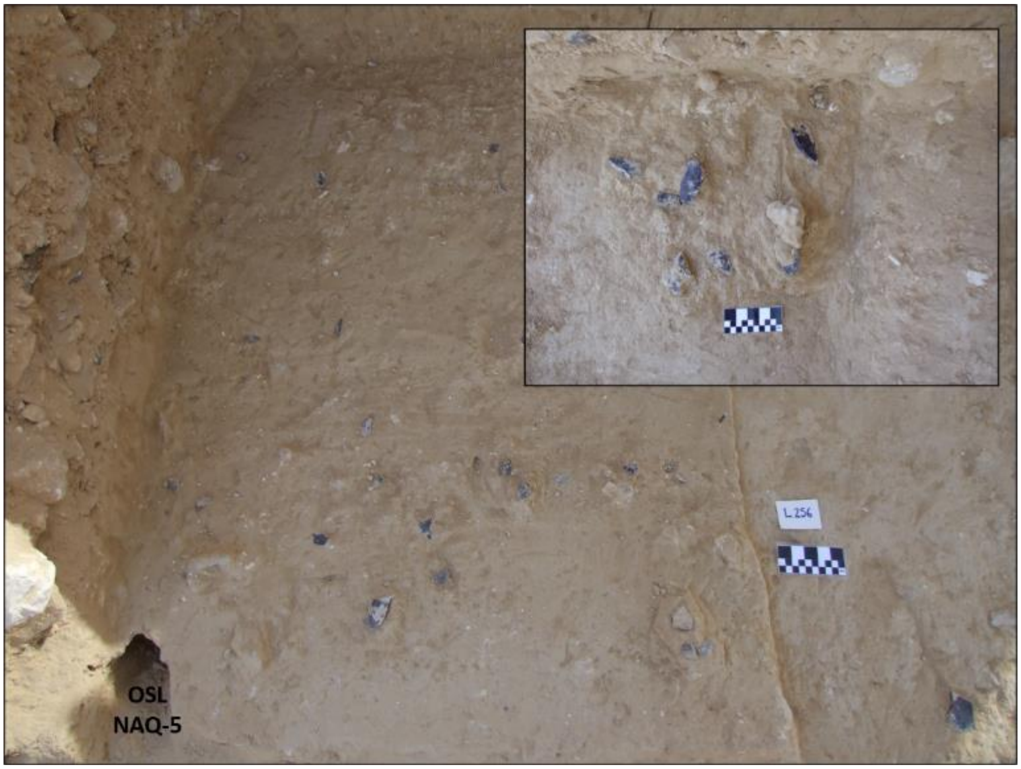

Figure S2.5. Exposure of lithic artifacts at the bottom of Unit 7.

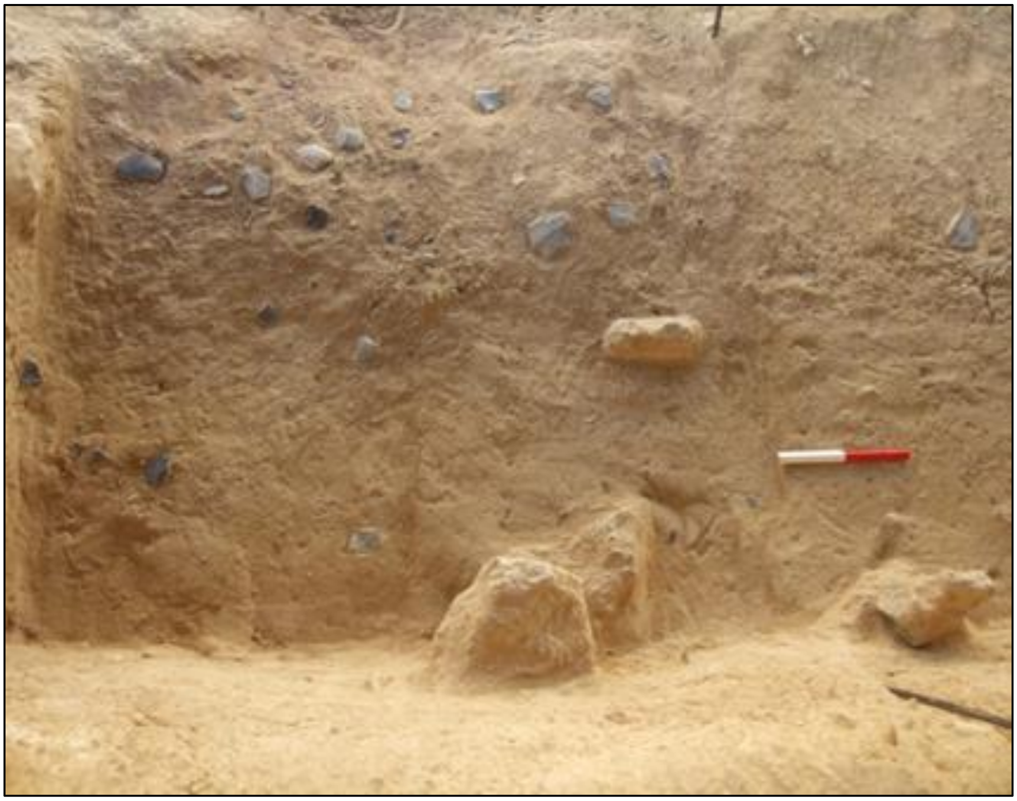

Figure S2.6. Exposure of lithic artifacts in Unit 11.

Section 3. Evidence for a hearth feature in Unit 11

We noted concentrations of flints in Unit 11, some of which were clearly fire-cracked and associated with dark local patches of sediments. FTIR analysis of the sediments in and around these dark patches showed that the dark patches contained substantial amounts of gypsum, calcite, clay and quartz, whereas the sediments around the patches contained only quartz, clay and calcite (Fig. S3.1). This raised the interesting possibility that the gypsum in these patches could actually result from fires where Tamarix wood was one of the fuel components. It is known that the ash of the wood of the Tamarix is composed of the calcium sulfate mineral, anhydrite (25-26) and the fresh wood contains crystals of calcium sulfate hemihydrate (basanite)(27). After burning these crystals presumably transform into anhydrite and anhydrite ash crystals would presumably undergo hydration over time to form gypsum, as was shown experimentally (28). Tamarix trees are abundant in the dry riverbed in the Nahal Aqev region today. We therefore carried out a series of experiments to examine this hypothesis.

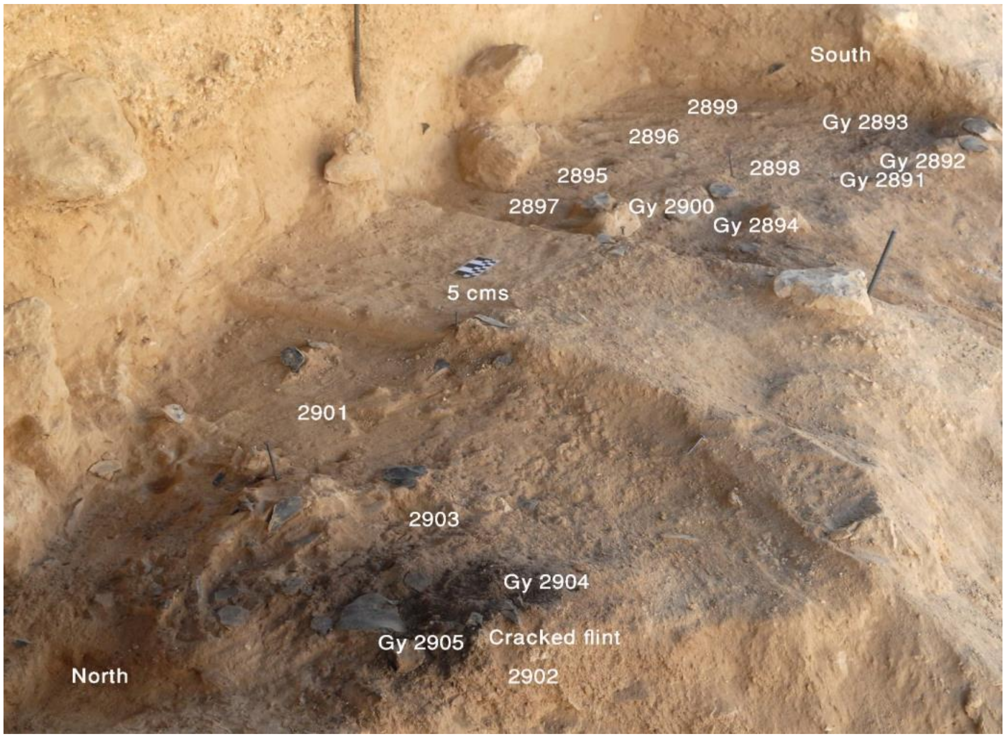

Figure S3.1. Unit 11 showing a concentration of flint debitage, some of which were cracked presumably due to exposure to fire. Samples numbers with the prefix Gy contain gypsum. These samples are located in sediments which were visually darker than the surrounding sediments.

Sediments with gypsum are present outside the site derived from various geological horizons. We examined the gypsum crystal morphologies in Unit 11 using a light microscope to determine whether their morphologies differed from those in the control sediments from outside the site. We could not identify any unique morphologies. It has been noted that the crystals in freshly prepared Tamarix ash do have characteristic rhombohedral shapes (28), but rhombohedral shaped crystals were not observed in Unit 11 sediments. We presume that the crystal shape changed either during the transformation from anhydrite to gypsum, or over time since burial. We then examined the clay in the FTIR spectra from 4 gypsum-rich sediments and 3 sediments around the gypsum-rich patches, all from Unit 11 (see Figure S3.1 for locations) (Fig. S3.2). The hydroxyl peaks of the clay spectrum located at 3691 and 3620cm^{-1} are very small or absent in the gypsum-rich samples but are small but prominent in the surrounding sediments. When clay is heated above around 500 °C (29) these hydroxyl peaks disappear. At higher temperatures the peak at 512cm^{-1} is diminished and the main peak at around 1032cm^{-1} shifts to higher wavenumbers. The clays in the gypsum-rich sediments do not show these features. We

therefore conclude that the clays associated with the gypsum were exposed to temperatures around 500 °C. This is therefore consistent with these gypsum-rich patches containing Tamarix ash and were hence hearths.

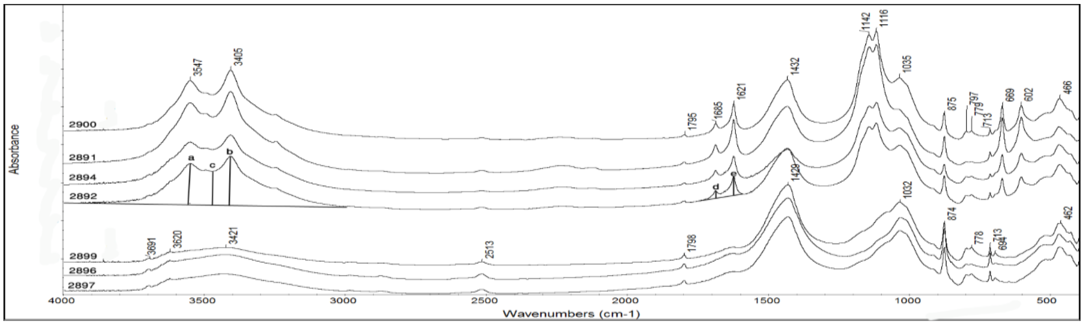

Figure S3.2. The infrared spectra of samples from the Unit 11 surface shown in figure 3.3. The top 4 spectra contain gypsum (prominent peaks at 3547, 3405, 1685, 1621, 1141, 1116, 669 and 602 cm^{-1}). Note that the small hydroxyl peaks of the clay minerals at 3691 and 3620 cm^{-1} are prominent in the 3 spectra at the bottom of the figure that do not contain gypsum, but are absent or barely visible in the spectra that do contain gypsum. This indicates that the sediments containing gypsum have been exposed to temperatures around 500°C. The spectra were all normalized to the same heights of the main 1032cm^{-1} peak of clay. The manner in which the parameters for the grinding curve of gypsum (see below) were obtained are shown in the spectrum of sample 2892. Parameter A is the heights of peaks (a) and (b) divided by the height of the lowest point between them (c). Parameter B is the height of peak (e) divided by the height of peak (d).

As calcite from wood ash is more disordered at the atomic level than geological calcite, we examined the possibility that the gypsum itself was more disordered at the atomic level even though it had diagenetically hydrated from anhydrite to gypsum. We therefore produced grinding curves for the pure gypsum control samples from outside the site by plotting parameter A against parameter B as defined in the legend of Figure S3.2. Figure S3.3 compares the grinding curves of the gypsum in the dark patches in Layer 11 to the gypsum-rich geological controls. All the gypsum-rich sediment samples from Unit 11 fall slightly above the 3 control curves derived from geological gypsum from this area. This may indicate that they are less well ordered. However, the gypsum from two of the control sediments taken from the top of the terrace about 100 meters south of the site, also had the same degree of disorder. Thus, degree of atomic disorder is not unique to the gypsum in the dark patches from Unit 11. Note that we cannot determine the atomic disorder of freshly prepared Tamarix ash, as it is anhydrite and not gypsum. As noted, over time the anhydrite can be expected to hydrate and become gypsum (28).

Thus, the observations consistent with the gypsum in these patches originating from hearths in which Tamarix wood was burned are: 1. The presence of localized patches surrounded by sediment without gypsum, which is consistent with these patches being hearths. 2. The absence or reduction of the clay hydroxyl peaks indicating heating of the sediments in the patches. 3. The association of the gypsum-rich samples with fire-cracked flint debitage, indicating that fires were made on the Unit 11 surface.

Patches of gypsum-rich sediments are also present in Unit 7. Unit 7 contains abundant flint debitage, some of which can be refitted. It is therefore clearly an occupation layer, and the dark gypsum-rich patches in Unit 7 may also be remnants of hearths.

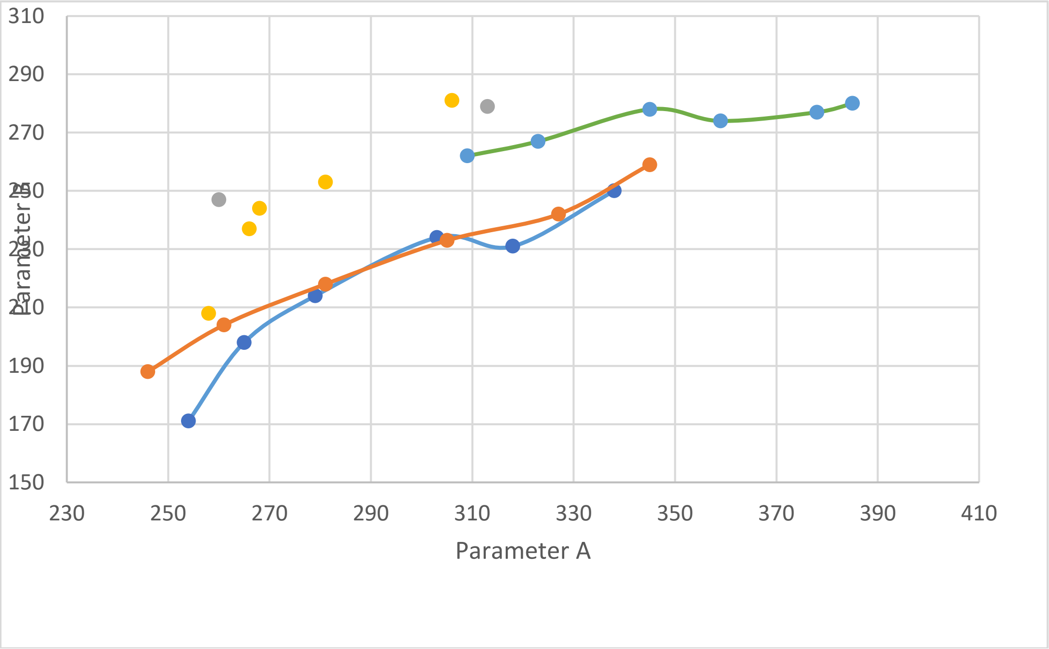

Figure S3.3. Gypsum grinding curves. Parameter A (X-axis) is plotted against Parameter B (Y-axis). These two parameters are defined in figure S3.2. Blue curve: large gypsum crystals from an area about 20kms from the site. Brown curve: fine grained powdery sample containing gypsum and sponge spicules from about 300m south of the site of Nahal Aqev. Green curve: gypsum crystals from a crack in the limestone bedrock about 250m south of the site of Nahal Aqev. Yellow data points: gypsum-rich samples from Unit 11. Gray data points: control sediments from about 100m south of Nahal Aqev.

Section 4. The lithic assemblages

The lithic assemblages from the renewed excavation comprise 17,804 artifacts. Of these 6,656 (37.4%) are larger than 2 cm (S4 Table 1). The artifacts were retrieved from the different excavated archaeological units, as well as surface and section cleaning. Almost all flint items are

extremely fresh and well preserved, including those from sections and surface materials. For the samples subjected to attribute analysis (N=2861), 97% of items were fresh and 3% slightly abraded. High number (55%) of the sample items were broken, partially due to fire, mainly in Unit 11.

Most of the flint items (81% of analyzed sample) were made using brown and grey raw material, probably from the Early Eocene Mor Formation cropping out around the site (30). Few items (5%, and none of the cores) were made using semi-translucent brown flint, most likely from the Mishash Formation that can be found ~2 km to the north of the site (5) or collected from gravels found along the main channel of the Aqev stream. Other flint types are very rare or cannot be assigned to a specific geological source.

All *in situ* layers show a high percentage of artifacts smaller than 2 cm (chips, 62.6%). In the upper Units 7 and 9 the high proportion of small artifacts seems to be the outcome of knapping on site. In Unit 11 the high proportion of small artifacts clearly also results from burning and flint bursting/cracking by fire.

S4 Table 1. General breakdown of the lithic assemblages by units at Nahal Aqev.

	Unit 7		Unit 9		Unit 11		Sections & trenches		Total	
**Type**	**N**	**%**	**N**	**%**	**N**	**%**	**N**	**%**	**N**	**%**
Primary elements	517	5.75	23	4.3	150	3.1	304	8.95	994	5.58
Flakes	1,958	21.77	81	15.1	647	13.3	868	25.57	3,554	19.96
Blades	140	1.56	1	0.2	21	0.4	66	1.94	228	1.28
Bladelets	77	0.86	0	0.0	14	0.3	10	0.29	101	0.57
Levallois Flakes	304	3.38	8	1.5	83	1.7	142	4.18	537	3.02
Levallois Blades	31	0.34	0	0.0	10	0.2	45	1.33	86	0.48
Levallois Points	17	0.19	1	0.2	2	0.0	48	1.41	68	0.38
Kombewa flakes	3	0.03	0	0.0	0	0.0	1	0.03	4	0.02
NBK	8	0.09	0	0.0	3	0.1	3	0.09	14	0.08
CTE	86	0.96	6	1.1	26	0.5	46	1.35	164	0.92
Spalls	5	0.06	0	0.0	0	0.0	0	0.00	5	0.03
**Total Debitage**	**3,146**	**34.98**	**120**	**22.4**	**956**	**19.6**	**1,533**	**45.15**	**5,755**	**32.32**
Chunks	198	2.20	13	2.4	150	3.1	208	6.13	569	3.20
Chips	5,445	60.55	396	73.9	3,735	76.5	1,572	46.30	11,148	62.62
**Total Debris**	**5,643**	**62.75**	**409**	**76.3**	**3,885**	**79.6**	**1780**	**52.43**	**11,717**	**65.81**
**Cores**	**70**	**0.78**	**5**	**0.9**	**14**	**0.3**	**49**	**1.44**	**138**	**0.78**
**Tools**	**134**	**1.49**	**2**	**0.4**	**25**	**0.5**	**33**	**0.97**	**194**	**1.09**
**Total**	**8,993**	**100.00**	**536**	**100.0**	**4,880**	**100.0**	**3,395**	**100.00**	**17,804**	**100.00**

Technology and waste

The debitage in all assemblages is dominated by flakes (61.7% of the debitage). Primary elements and Levallois blanks are present in moderate percentages (17.3% and 12.1% respectively) while cores, core trimming elements (CTE) and blades occur in low frequencies. The dominant reduction sequence in all assemblages is centripetal Levallois in recurrent mode. This tendency is seen on flakes and Levallois blank frequencies as well as on cores (S4 Table 1) (Figs. S4.1-2; 4-5).

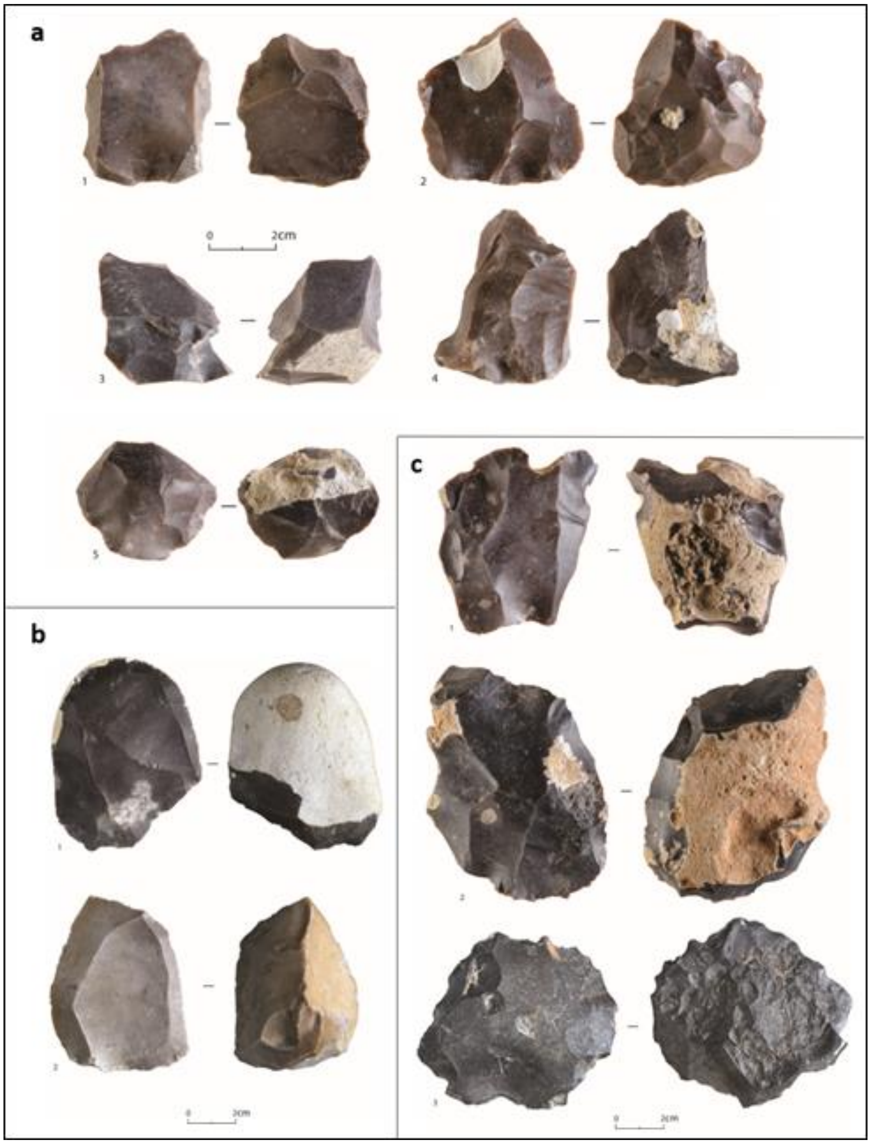

Figure S4.1. Levallois Cores: a: cores from Unit 7: 1-4- exhausted Levallois cores for flakes, 5- Core on flake. b: cores from upper sections 1-2- Levallois core for points. c: cores from Unit 11: 1- bidirectional Levallois core, 2-3- centripetal Levallois core for flakes (burnt).

In the assemblage of Unit 11 the centripetal Levallois reduction sequence seems to be almost the only one present, except for some Levallois blanks showing bidirectional flaking, mostly on elongated items. In the upper units and in the surface material more technological variability is apparent. This is expressed by the presence of unidirectional convergent Levallois flaking as well as higher frequencies of non-Levallois flaking methods for flakes and bladelet production (mainly single platform cores and core on flakes). Levallois points are present mostly in the upper units, but in most cases do not show the "classic" triangular morphology with Y scar pattern.

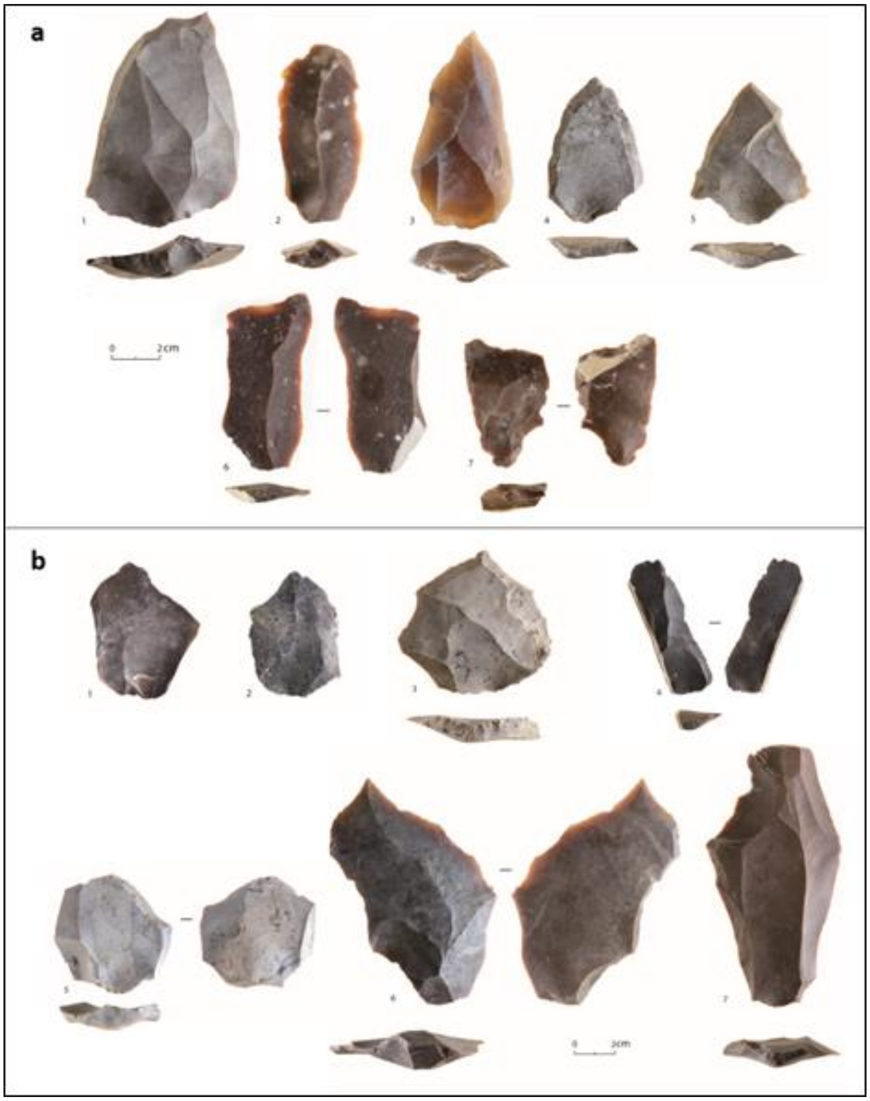

Figure S4.2. Levallois debitage: a: Unit 7: 1-Levallois flake, 2- Levallois blade, 3-5- Levallois points, 6-7- CTE debordant. b: Unit 11: 1-3- Levallois flakes, 4-NBK, 5-6- CTE debordant, 7- CTE Overshot.

Nevertheless, centripetal knapping strategy is the most prominent in the whole assemblage, as seen on items with identifiable scar pattern (Figs. S4.4-5), both Levallois and non-Levallois, probably partly derived from the same reduction sequence. Cores constitute 2.9% of the flint items >2 cm in the assemblage, with a lower frequency (1.2%) in Unit 11. Levallois cores are the largest group within the core types in all assemblages except for Unit 9, where the small sample size may have affected the composition (Fig. S4.3). Centripetal cores are clearly the most common type of the Levallois cores in all units (Fig. S4.4).

The hierarchical surface cores in the assemblage are most likely very exhausted Levallois cores. Other frequent core types are Core on Flakes and single platform cores; the latter characterize the upper units but are absent from Unit 11. Half of the cores in the assemblage were found in an exhausted stage, maybe due to limited nodule size and not lack of raw material near the site. This can maybe also be seen in core metric attributes. Maximum length of cores in Nahal Aqev range between 128-25 mm (average 61.6 mm) and are larger than average in Unit 11.

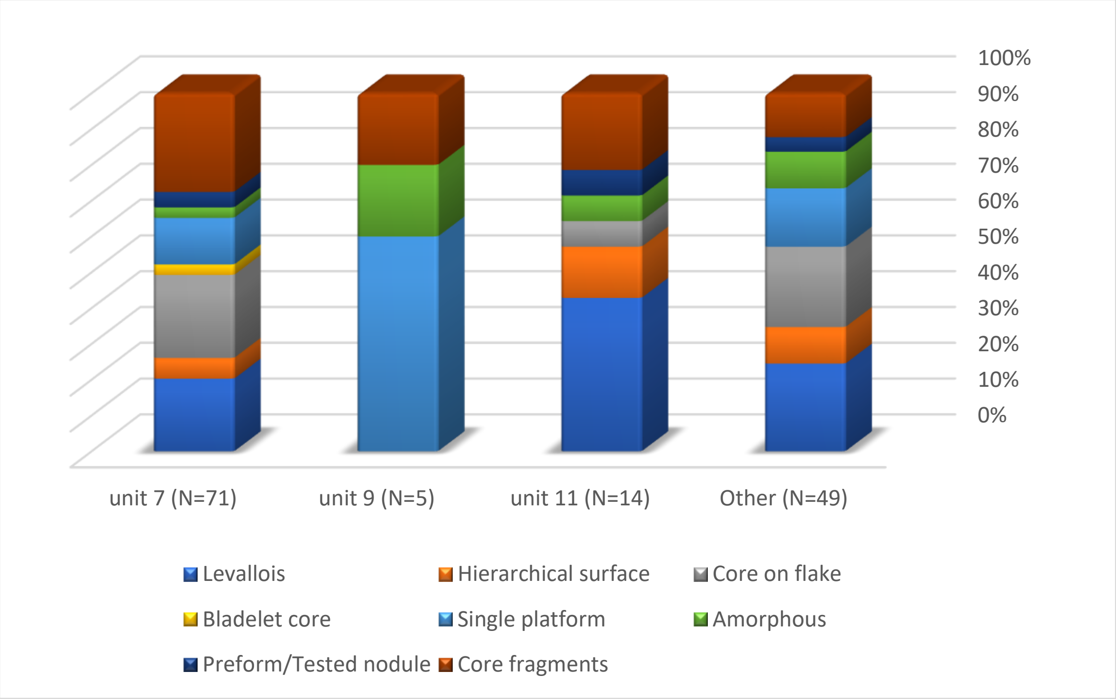

Figure S4.3. General breakdown of cores by units.

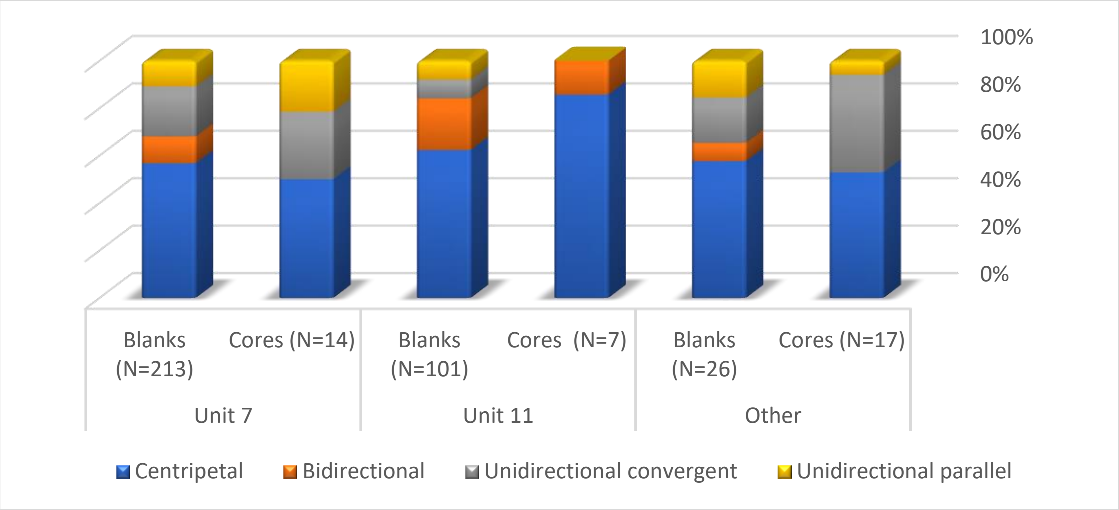

Figure S4.4. Scar patterns on Levallois cores and blanks. cores include hierarchical surface cores and blanks include retouched and unretouched items (broken items with indeterminate scar pattern are not included in the graph).

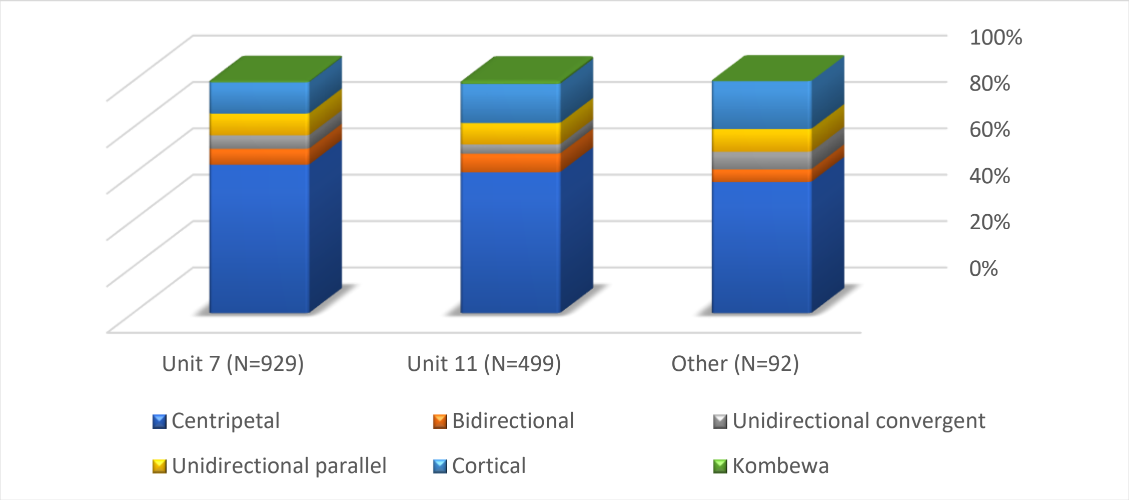

Figure S4.5. scar patterns on all blanks (Levallois and non-Levallois) when identifiable.

Blanks modified by retouch constitute 2.9% of the flaked items >2 cm (0.78% of the total assemblage) and are dominated by retouched items and notched and denticulated items (S4 Table 2; Fig. S4.6). Levallois blanks were frequently chosen for modification (Fig. S4.7), and even more prominently in Unit 11. The tool assemblage of Unit 7 is larger and more varied, including a group of small and finely retouched awls and raclettes. The tools of Unit 11 are characterized with more intense and invasive retouch than in other units, both on side scrapers and retouched flakes.

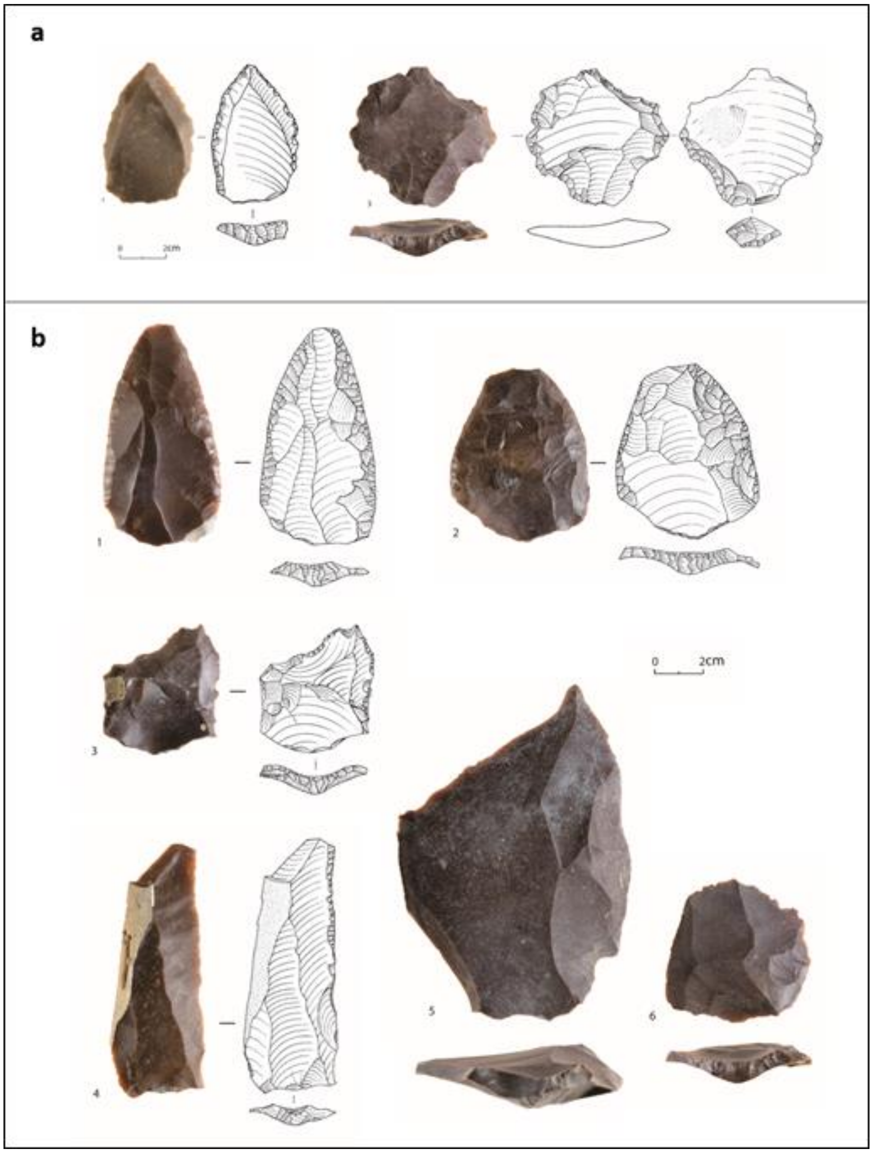

Figure S4.6. Retouched items: a: unit 7: 1- retouched Levallois point, 2- Denticulate b: unit 11: 1-2,5- side scrapers, 3-4,6- retouched Levallois items.

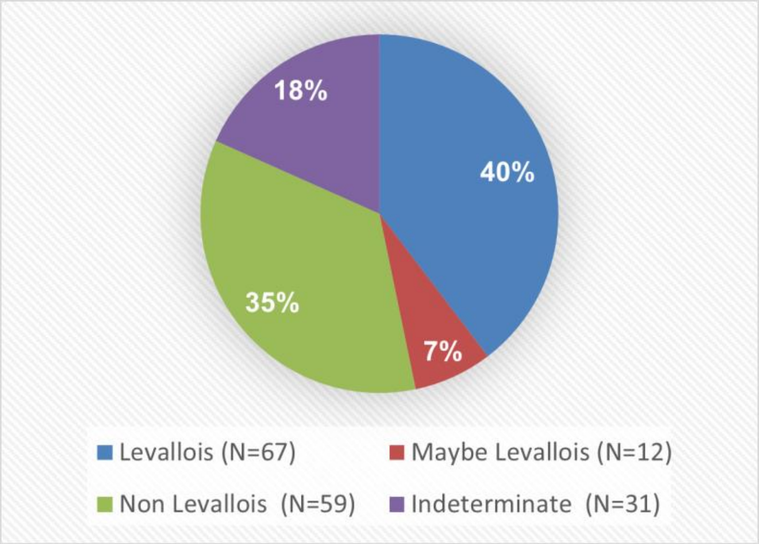

Figure S4.7. Technology of retouched blanks.

S4 Table 2. General breakdown of tools by units at Nahal Aqev.

	Unit 7		Unit 9		Unit 11		Sections & trenches		Total	
**Type**	**N**	**%**	**N**	**%**	**N**	**%**	**N**	**%**	**N**	**%**
Side scraper	3	2.2	0	0.0	2	8.0	1	3.0	6	3.1
Retouched Lev. Flake	36	26.9	1	50.0	11	44.0	4	12.1	52	26.8
Retouched Lev. point	4	3.0	0	0.0	0	0.0	1	3.0	5	2.6
Notch and Denticulate	22	16.4	0	0.0	1	4.0	11	33.3	34	17.5
Retouched Flake	42	31.3	0	0.0	7	28.0	10	30.3	60	30.9
Retouched Blade	7	5.2	0	0.0	1	4.0	1	3.0	9	4.6
Raclette	4	3.0	0	0.0	0	0.0	0	0.0	4	2.1
Borer and Awl	7	5.2	0	0.0	1	4.0	1	3.0	9	4.6
Burin	0	0.0	0	0.0	0	0.0	1	3.0	1	0.5
Truncated item	4	3.0	0	0.0	1	4.0	1	3.0	6	3.1
End Scraper	0	0.0	0	0.0	0	0.0	1	3.0	1	0.5
Massive tool	1	0.7	0	0.0	0	0.0	1	3.0	2	1.0
Tool fragment	4	3.0	1	50.0	1	4.0	0	0.0	5	2.6
**Total**	**134**	**100.0**	**2**	**100.0**	**25**	**100.0**	**33**	**100.0**	**194**	**100.0**

In summary, lithic assemblages from all units excavated in Nahal Aqev share some general attributes, but also differ from one another in some technological aspects. The main differences are between the assemblage from the lower most layer Unit 11 and the rest of the units. Unit 11 is less varied technologically and typologically, and the dominance of the Levallois centripetal flaking method is more prominent in this unit.

Section 5. Luminescence dating

Ten samples were collected in 2016 from the exposed sections, from the archaeological units and from above and below them (S5 Table3). An additional sample was collected in 2017 (NAQ-16) from the south section after it was pushed and excavated 1.5 m further to the south. The stratigraphic correlation of this sample with the units in the section samples previously, is not clear, nor is its exact burial depth. Although it definitely forms part of the terrace section, due to these uncertainties it is listed only in the SM table.

An auger with a diameter of 2" was used for drilling horizontally into the excavated sections and extracting the sediment. The exterior 1-15 cm and interior 30-40 cm were used for evaluating dose rates as two separate samples, to account for the sediment inhomogeneity, while the sediment taken from 15-30 cm depth was used for extracting quartz and alkali feldspar grains. Sampling was carried out under an opaque tarpaulin to prevent any exposure to sunlight, and samples were placed immediately into light-tight black bags .

The source of the quartz in the sediment is aeolian, and all surrounding bedrock is limestone. The quartz was blown in during dust storms and is deposited on the surface, and later washed into the streams and deposited in the terraces. On average modern dust contains ~30% quartz in the very-fine-sand to fine-silt size fractions, and in the past dust had a similar grain size distribution and composition (31-32).

Quartz in the size range of 90-125 µm was extracted and purified using routine laboratory procedures (10). Briefly, after wet sieving to the desired grain size, carbonates were dissolved with 8% hydrochloric acid (HCl). The rinsed and dried sample was passed through a Frantz magnetic separator (33) to remove any undissolved dolomite, heavy minerals, and some feldspars. Three grams of the non-magnetic fraction were etched for 40 minutes in hydrofluoric acid (40%) to dissolve the remaining feldspars and etch the exterior of the quartz grains, followed by soaking overnight in 16% HCl to remove any fluorides that may have precipitated. This was followed by rinsing and drying.

For seven samples, alkali feldspar (KF) grains were extracted from ~5 gr of the non-magnetic fraction (un-etched) using a one-step density separation with sodium polytungstate with a density of 2.58 gr/cm^3, followed by light etching with 10% HF for 10 min (34), which removed ~10 µm of the grains' surfaces.

The equivalent dose (De) values were measured using single aliquot regenerative dose (SAR) protocols (35) on TL/OSL Risø readers models DA-15 with a single grain attachment, DA-20

(DASH), or a refurbished DA-12, all equipped with calibrated ^{90}Sr beta sources. Each reader and sample carrier ("disc") type was calibrated using Risø calibration quartz (36), and dose rates to sand-size grains on aluminum discs in the three readers were 0.036, 0.109 and 0.043 Gy/s, respectively. Stimulation for multi-grain quartz OSL was by an array of blue LEDs (470 nm) delivering 37-59 mW/cm2 to the sample (depending on the reader model). For single grains (SG), OSL stimulation was by a green laser. Detection for both multi-grain and single grain quartz was through 7.5mm U-340 filters. KF grains were stimulated by IR diodes (870 nm) delivering 126-144 mW/cm2 to the sample (depending on the reader model) and detection was through a combination of Schott BG-39 and Corning 7-59 filter pack.

Measurement protocols are listed on S5 Table 4. For quartz, De was measured on 1-mm aliquots (multi-grain; MG) with ~50 grains per aliquot, mounted on aluminum discs. SG were measured in standard single grain discs with an array of 10x10 holes, each with a diameter and depth of 300 µm. The latter are effectively micro-aliquots, as hole diameter is 300 µm whereas grain size was 90-125 µm, such that in each grain hole there were 3-4 grains (37). Twenty-five multi-grain aliquots and 400-500 grain holes were measured for each sample. Micro-aliquot data were screened for further data processing using criteria defined in Porat et al. (38).

To obtain the best measurement conditions, a dose recovery test was carried out for sample NAQ-16. Twelve aliquots were bleached for two hours under natural sunlight and then bleached again in the reader for 100 s using blue diodes, followed by administering a laboratory dose of 78 Gy. This dose was treated as an unknown and measured using the SAR protocol (S5 Table4) under a range of preheat and cutheat conditions. A recovery of 0.95 (the ratio between measured and given dose) was obtained using a preheat temperature of 260°C, a test dose of 8 Gy and a cutheat temperature of 200°C (Fig. S51). These conditions were further used for all quartz measurements.

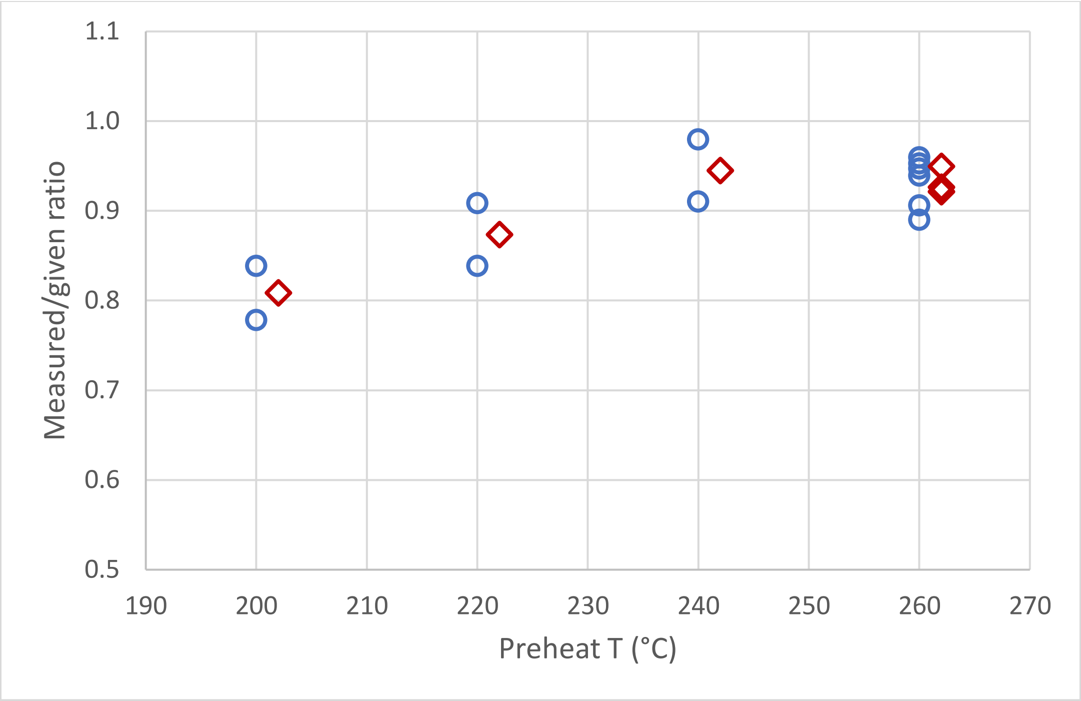

Fig. S5.1: Dose recovery results over a range of preheat (PH) and cutheat (CH) temperatures for sample NAQ-16. Blue circles – individual measurements; brown diamonds – averages. Three CH temperatures, at 200°C, 220°C and 240°C, were tested for the PH temperature of 260°C. For the remaining PH temperatures, CH was 20°C lower. The highest average recovery ratio, 0.95, was obtained with PH and CH temperatures of 260°C and 200°C, respectively.

For KF, De was measured on 1-mm aliquots (~50 grains) using stainless steel cups; due to very small amounts of separated KF, only 6 aliquots were measured for each sample. The post-infrared (IR) signal measured at an elevated temperature (pIR-IR) and the SAR protocol were used for De determination (39). The preheat and post-IR measurement temperatures, 280°C and 250°C, respectively, were selected after these were found to have the best tradeoff between ease of bleaching of the pIR-IR signal and anomalous fading in samples with a similar geological source (40). Anomalous fading was measured for four samples for which enough purified KF remained after De measurements (six aliquots each) for up to 50 h, following Buylaert et al. (41). The obtained g-values (S5 Table 3) were used to correct the KF ages for fading using the method of Huntley and Lamoth (42).

Average De values were calculated using essentially all measurements and the central age model, which assumes that the De values are distributed around a central value (43), with 1 sigma standard errors.

Dose rates were calculated from the concentrations of the radioactive elements U, Th and K. Sediment samples were dried, crushed, homogenized and split, and a 50-gr subset was powdered for further chemical analyses. The two sediment samples collected from either side of the luminescence samples were treated and measured separately, however their results were averaged for dose rate calculations. U and Th contents were measured by inductively coupled plasma (ICP) mass spectrometry (MS) and K by ICP optical emission spectrometry (OES). Relative errors of 10% for all measurements take into account the non-homogenous sediment. The KF internal K-content was estimated at 12.0 ± 0.5%. An a-value of 0.15 ± 0.05, an average of values given by Balescu et al. (44) and Rendell et al. (45) for sand-size KF, was used to calculate external alpha dose rates to the KF. Cosmic dose rates were evaluated from current burial depths and time averaged moisture content was estimated at 5% or 8% for samples shallower or deeper than 3 m, respectively. Attenuation factors used for dose rate calculations are from Nambi and Aitken (46).

S5 Table 3: Luminescence data and ages for Nahal Aqev samples.

Notes: Grain size for all samples – 90-125µm; moisture contents – 5% or 8% for samples shallower or deeper than 3 m, respectively; a-values for quartz and alkali feldspar (KF) – 0.1±0.02 and 0.15±0.05, respectively; internal K-content for KF – 12±0.5%, contributing an internal dose rate of 0.36 Gy/ka (not in Table). An additional ~0.15 Gy/ka is the alpha dose contributed by the milder HF etching of the KF. The K, U and Th contents are averaged from analyses of two separate sediment samples taken from either sides of the sample used to extract the quartz and KF; associated errors are ±10% of the value. MG –multigrain; SG –single grains; KF – alkali feldspar; KF* - alkali feldspar ages corrected for fading. For samples where no fading rates are given, the average of all measured samples, 1.3±0.2 %/decade, was used.

Lab code	Unit	Depth (m)	Mineral	Tech-nique	K (%)	U (ppm)	Th (ppm)	Fading (% /decade)	Alpha (Gy/ka)	Beta (Gy/ka)	Gamma (Gy/ka)	Cosmic (Gy/ka)	Dose Rate (Gy/ka)	Aliquots /grains	OD (%)	De (Gy)	Age (ka)
NAQ-1	Lower Unit 2	1.2	Quartz	MG	0.43	4.56	2.77		0.01	0.92	0.71	0.18	1.82±0.09	23/25	39	77±5	42±3
			Quartz	SG					0.01	0.92	0.71	0.18	1.82±0.09	79/80	76	78±7	42±4
NAQ-2	Unit 3	1.55	Quartz	MG	0.38	3.75	2.56		0.01	0.78	0.60	0.17	1.57±0.08	22/25	35	138±7	88±6
			Quartz	SG					0.01	0.78	0.60	0.17	1.57±0.08	99/100	82	101±9	67±7
NAQ-16	Unit 4	1.8	Quartz	MG	0.48	4.38	3.23		0.01	0.94	0.73	0.16	1.83±0.09	27/27	40	97±8	53±5
NAQ-3	Upper Unit 5	1.95	Quartz	MG	0.47	4.08	2.52		0.01	0.88	0.66	0.17	1.71±0.05	22/25	58	132±10	77±6
			Quartz	SG					0.01	0.88	0.66	0.17	1.71±0.05	73/73	62	107±8	63±5
			KF	MG				1.3±0.72	0.14	0.89	0.66	0.17	2.21±0.09	6/6	6	188±5	85±4
			KF*														96±6
NAQ-4	Lower Unit 5	2.3	Quartz	MG	0.41	3.42	2.06		0.01	0.75	0.55	0.16	1.47±0.07	25/25	30	144±9	98±8
			Quartz	SG					0.01	0.75	0.55	0.16	1.47±0.07	136/137	43	127±5	86±6
			KF	MG				1.5±0.28	0.12	0.75	0.55	0.16	1.95±0.10	6/6	6	207±6	107±6
			KF*														124±8
NAQ-6	Lower Unit 5	2.4	Quartz	MG	0.44	4.7	2.8		0.01	0.94	0.73	0.16	1.84±0.05	24/25	34	121±8	66±5
			Quartz	SG					0.01	0.94	0.73	0.16	1.84±0.05	120/120	53	136±7	74±5
			KF	MG				1.0±0.24	0.16	0.95	0.73	0.16	2.36±0.12	6/6	6	199±6	84±5
			KF*														92±6
NAQ-5	Unit 7	3.0	Quartz	MG	0.44	3.33	2.41		0.01	0.76	0.57	0.15	1.48±0.06	25/25	40	145±12	98±9
			Quartz	SG					0.01	0.76	0.57	0.15	1.48±0.06	146/147	44	137±6	92±6
			KF	MG				1.4±1.4	0.12	0.77	0.57	0.15	1.96±0.10	6/6	0	222±3	114±6
			KF*														131±23
NAQ-7	Unit 9	3.7	Quartz	MG	0.35	3.33	2.96		0.01	0.69	0.55	0.13	1.39±0.05	25/25	30	134±9	97±7
			Quartz	SG					0.01	0.69	0.55	0.13	1.39±0.05	56/62	50	190±11	137±9
			KF	MG					0.12	0.70	0.55	0.13	1.86±0.09	6/6	5	216±5	116±6
			KF*														132±7
NAQ-8	Unit 11	4.5	Quartz	MG	0.77	2.73	4.46		0.01	0.92	0.65	0.12	1.70±0.08	25/25	37	140±11	83±8
			Quartz	SG					0.01	0.92	0.65	0.12	1.70±0.08	69/70	50	139±9	82±7
			KF	MG					0.11	0.93	0.65	0.12	2.17±0.11	6/6	4	225±5	103±6
			KF*														117±7
NAQ-9	Base of Unit 11	5.0	Quartz	MG	0.66	2.74	4.06		0.01	0.84	0.61	0.12	1.58±0.07	23/25	45	167±12	106±9
			Quartz	SG					0.01	0.84	0.61	0.12	1.58±0.07	176/177	56	148±7	94±6
			KF	MG					0.11	0.85	0.61	0.12	2.04±0.10	6/6	0	261±4	118±6
			KF*														134±7
NAQ-11	Unit 17	7	Quartz	MG	0.43	2.31	3.01		0.01	0.62	0.47	0.11	1.21±0.056	22/25	37	125±7	106±8
			Quartz	SG					0.01	0.62	0.47	0.11	1.21±0.056	62/64	63	138±11	116±11

S5 Table4: Luminescence protocols used to measure equivalent doses in this study. OSL (optically stimulated luminescence) multi-grain (MG) and single grain (SG) protocols modified from Wintle and Murray (47); pIR-IR_{250} (post-infrared stimulated luminescence at 250°C) protocol modified from Thiel et al. (2011), which includes both IR_{50} and $pIRIR_{250}$ (p stands for post). The signals used for constructing a dose response curve (DRC) and calculating the De are shaded.

	**a. OSL MG**	**b. OSL SG**	**c. pIR-IR_{250}**
Holder type	Aluminum discs	10 by 10 300 µm holes stainless steel discs	Stainless steel cups
Aliquot size	1 mm; ~50 grains	3-4 grains	1 mm; ~50 grains
Integration for DRC	Signal: 0.2 s<br>Background: 2 s	Signal: 0.1 s<br>Background: 0.2 s	Signal: 0.6 s<br>Background: 3 s
Step			
1	Give a regen dose (for N dose = 0)	Give a regen dose (for N dose = 0)	Give a regen dose (for N dose = 0)
2	Preheat for 10 s @ 260°C	Preheat for 10 s @ 260°C	Preheat for 60 s @ 280°C
3	Measure OSL with LED for 40 s @ 125°C (Ln)	Measure OSL with green laser for 1 s @ 125°C (Ln)	Measure IRSL for 200 s @ 50°C
4	Give a test dose of 9 Gy	Give a test dose of 21 Gy	Measure IRSL for 200 s @ 250°C (Ln)
5	Preheat for 5 s @ 200°C	Preheat for 5 s @ 200°C	Give a test dose of 32 Gy
6	Measure OSL with LED for 40 s @ 125°C (Tn)	Measure OSL with green laser for 1 s @ 125°C (Tn)	Preheat for 60 s @ 280°C
7	Deplete remaining OSL for 100 s @ 280°C	Deplete remaining OSL for 100 s @ 270°C	Measure IRSL for 200 s @ 50°C
8	Return to Step 1	Return to Step 1	Measure IRSL for 200 s @ 250°C (Tn)
9			Deplete remaining IRSL for 200 s @ 325°C
10			Return to Step 1

Luminescence ages

S5 Table 3 and Fig S5.2 present the ages obtained for the eleven measured samples using quartz multi-grains and single grain (micro aliquots) measurements, and the seven samples measured by KF multi-grains and corrected for fading. The SG quartz ages range from 42±4 ka at the top (Unit 2) to 115±11 ka at the bottom (Unit 17); the multi-grain ages range from 42±3 ka to 104±8 ka for the same units; the KF pIR-IR_{250} ages range from 85±4 ka (unit 5) to 118±6 ka for (unit 11); while the fading-corrected KF age range from 92±6 ka to 137±7 ka for units 5 to 11, respectively. For all samples, the natural OSL and pIR-IR_{250} signals are bright and decay fast (Fig. S5.3 a-c), recycling ratios on dose response curves are close to unity and recuperation is negligible (Fig. S5.3 d-f). For about half the samples, particularly in the lower parts of the section, the OSL De values are close to the $2D_0$ typical of quartz from this region, ~140 Gy (48), so possibly these quartz ages might be underestimated. The dose distributions are mostly normal (Fig. S5.3 g-i), justifying the use of the central age model for calculating the significant average De and ages.

The quartz OSL multi-grain measurement have medium to high over-dispersion (OD) values, 30-58% (S5 Table 3). These most likely result from the small size of the aliquots, whereby within the ~50 grains on the disc only very few (1-5) grains have a bright signal (49). Another possible source for scatter is from some spatial heterogeneity in the beta dose field, the result of the coarse nature of the sediment (abundant limestone gravels and, in places, also limestone cobbles). This limestone is generally poor in radioactive elements when compared to the finer matrix which is mostly derived from loess and is more clay-rich. The normal De distribution suggests that partial bleaching at the time of deposition was not significant, also implied by the lack of a significant number of outlying aliquots with high De values (Fig. S5.3 g). Also the quartz SG De distributions do not indicate partial bleaching of the grains (Fig. S3 h). Fading rates measured for the KF samples are low and similar among samples, averaging 1.3±0.5 % per decade.

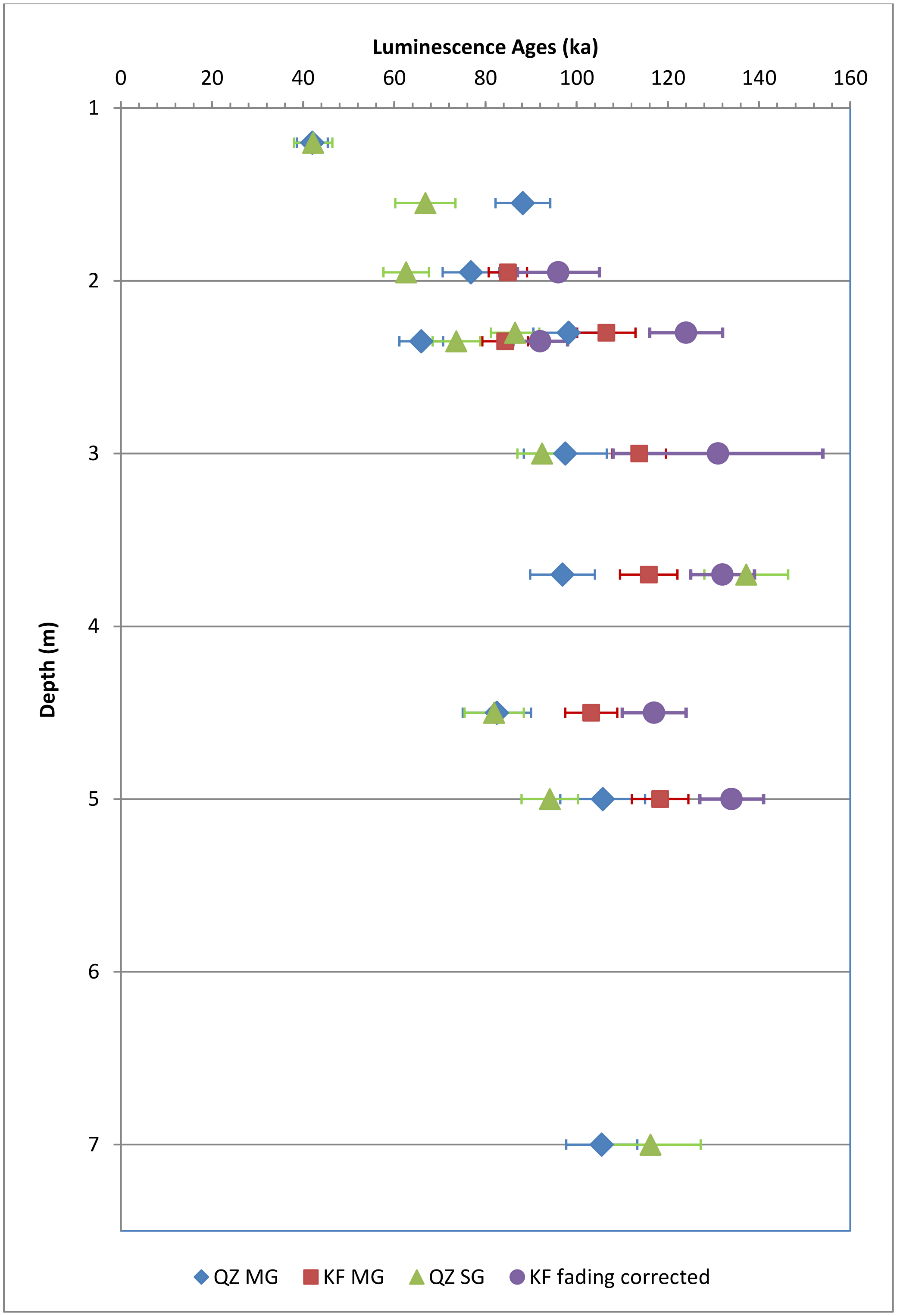

Fig. S5.2: Luminescence ages as a function of depth in the sediment profile for the different minerals and protocols. QZ – quartz; KF – alkali feldspar; MG – multi-grain; SG – single grains.

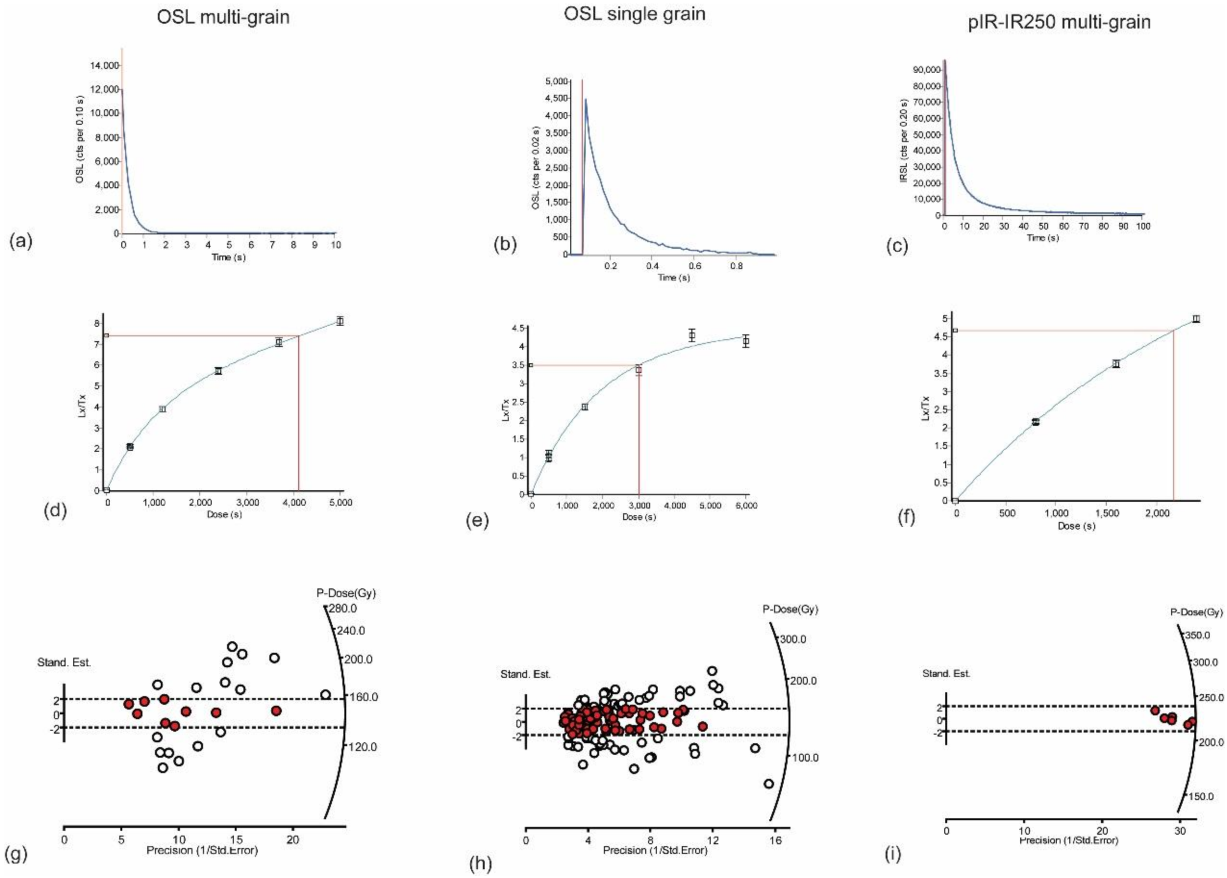

Fig. S5.3: Luminescence results for sample NAQ-5. Aliquot size, stimulation source and integration channels are listed in S5 Table 4. (a) Natural OSL (blue LED stimulation) signal for one quartz multi-grain aliquot (~50 grains), showing the first 10 s (of 40 s), displaying the rapid decay of the signal. (b) Natural OSL (green laser stimulation) signal for one quartz micro-aliquot (3-4 grains), measured for 1 s. (c) Natural pIR-IR$_{250}$ (IR stimulation) signal for one aliquot of alkali-feldspar grains (~50 grains), showing the first 100 s (of 200 s). (d) Dose response curve for the same aliquot as in (a), De = 178±11 Gy. The data points were fitted using an exponential+linear fit. The recycling ratio (RR; the repeated measurement point at 21 Gy) = 0.97, IR depletion ratio = 0.98, recuperation = 0.6% and D0 = 78 Gy. (e) Dose response curve for the same grain hole as in (b), De = 134±14 Gy. The data points were fitted using an exponential+linear fit. RR = 0.91, IR depletion ratio = 0.93, recuperation = 0.6% and D0 = 86 Gy. (f) Dose response curve for the same aliquot as in (c), De = 216±7 Gy. The data points were fitted using an exponential fit. RR = 1.01, recuperation = 0.2% and D0 = 268 Gy; De is not sensitive to the selected channels. (g) Radial plot for all 25 measured quartz multi-grain aliquots. Average De, calculated using the central age model (CAM), is 145±12 Gy and over-dispersion (OD) = 40%. (h) Radial plot for all 146 accepted micro-aliquot measurements. Average De, calculated using CAM, is 137±6 Gy and OD = 43%. (i) Radial plot for all 6 measured alkali feldspar multi-grain aliquots. Average De, calculated using the CAM, is 222±3 Gy and OD = 0%.

The average ratio between the quartz SG ages and the corresponding multigrain ages is 1, but if we disregard sample NAQ-7 (at 3.6 m), whose SG age is ~40% older than the multi-grain age, then the SG ages are on average ~5% younger than the multigrain ages (S5 Table 3; Fig. S5.2). This can be explained by the criteria used for selecting valid SG De values, whereby grains with a high natural signal that cannot be regenerated by the laboratory beta dose are discarded; in multigrain measurements these contribute to the natural signal and De.

The uncorrected KF ages are older than the multi-grain quartz ages by 8-21 ka, and the difference increases with depth. This difference probably indicates that for the older samples, the quartz De values are nearing saturation and their ages are thus somewhat underestimated. Indeed, plotting the De values as a function of depth shows that while the De values of the quartz hardly increase below 2 m, those of the KF do increase with depth (Fig. S5.4). Thus, we consider the pIR-IR_{250} KF ages to be more reliable. Measured fading rates are low, averaging 1.3±0.2 %/decade. For such low values, corrections are often considered unnecessary (50). However, corrections increase the KF ages by 8 to 17 ka, depending on the specific fading rate and uncorrected age; they also increase the errors on the ages, particularly for samples with large errors on the fading rates. Nonetheless, in the case of Nahal Aqev, the corrected KF ages agree better with the age obtained from micro-tephra analyses (see below).

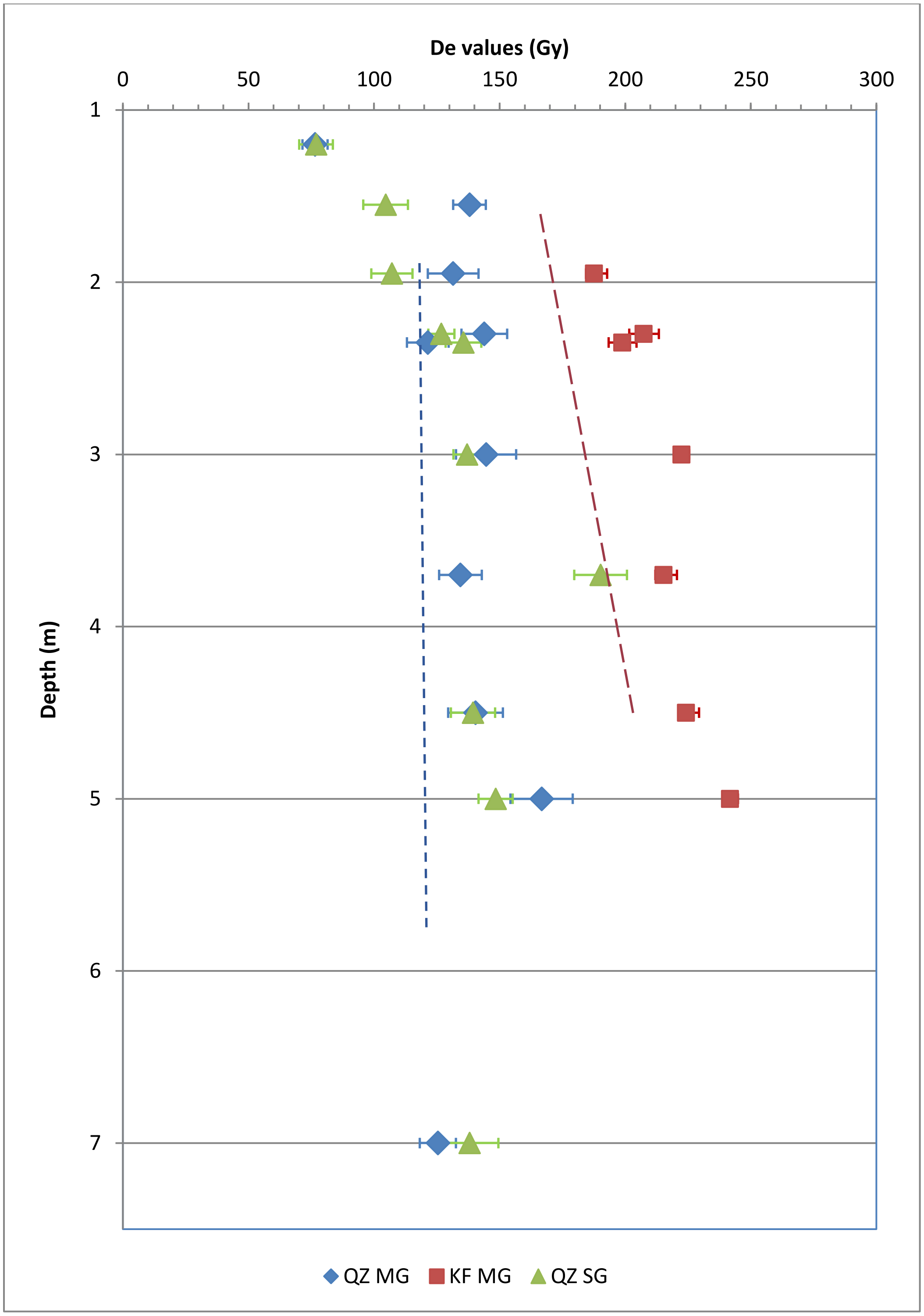

Fig. S5.4: A plot of De values as a function of depth in the sediment profile. Note that the quartz MG De values do not increase below ~2. m, whereas the feldspar De values continue growing.

Overall, the ages increase with depth up to ~ 2.3 m, and below that are almost constant (Fig. S5.2). As the De values of the KF continue to increase with depth (Fig. S5.4), we have no reason to suspect that the pIR-IR_{250} ages are in saturation; rather the case here is of rapid sedimentation.

The ages increase with depth up to ~ 2 m, and below that are almost constant. One outlier is the paired samples NAQ-4 and NAQ-6, both from the same layer at 2.3 m depth but from different aspects of the excavation walls. It is not possible to assess which sample better represents the age of that unit, as (the younger) NAQ-6 is in agreement with the overlying sample NAQ-3, while (the older) NAQ-4 is within the range of the underlying sample NAQ-5. It is worth noting that the De values of those samples are similar for both quartz and KF (Fig. S4), suggesting that the dose rates of either samples might have not been estimated correctly.

With these considerations in mind and using the fading corrected pIR-IR_{250} KF ages, our best estimates for the ages of archaeological Units 7, 9 and 11 are as follows: Sample NAQ-5 gives an age of 131±23 ka for Unit 7; sample NAQ-7 gives an age of 132±7 ka for Unit 9; and samples NAQ-8 and NAQ-9 bracket Unit 11 to between 117±7 ka and 134±7 ka. Overall, the ages all fall within the very end of marine isotopic stage (MIS) 6 and early MIS 5. The ages for Unit 11 agree very well with the age obtained from the micro-tephra analyses of 126-128 ka from the same Unit (See Fig. 4 in main text).

Section 6. Cryptotephra

Cryptotephra sampling

Samples for cryptotephra analysis were collected at Nahal Aqev in September 2019 at 5 cm consecutive and contiguous intervals from existing and freshly cleaned profiles of the site (Fig. S6.1). A total of 41 bulk sediment samples (each ~50 g dry weight) were taken from 2 sampling columns within the new excavation area, with Column 1 spanning Unit 3 to Unit 7 extending from the top of the exposed section to a depth of 1.35 m. Columns 2/2A spanned a depth of 0.7 m extending from Unit 9 to Unit 11, with the upper 0.15 m of the column stepped into an oerlying profile immediately above.

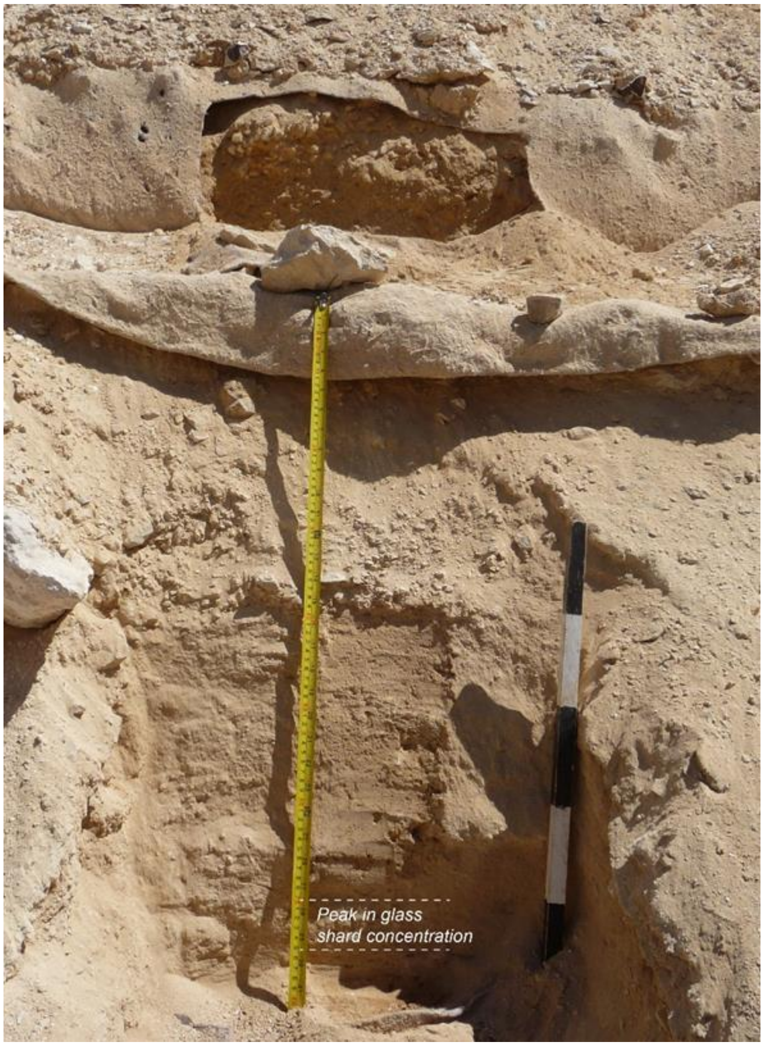

Figure S6.1: Column 2 sampling profile. The peak in glass shard concentration was identified at a relative depth of 70-75 cm.

Cryptotephra processing

In the laboratory, individual sediment sub-samples weighing ~2-3 g were extracted from the 41 bulk sediment samples, with three to four of these combined to form composite or 'scan' samples. 13 scan samples were produced to span the sampled Nahal Aqev sequence.

Scan samples were placed in an oven overnight at 105°C to establish their dry weight and combusted in a laboratory muffle furnace for two hours at 550°C to remove organic detritus. Residual material was immersed in 10% Hydrochloric acid (HCL) to dissolve carbonates before

being passed through nylon sieve meshes with apertures of 125 and 15 µm. All material in this size fraction was retained for further processing.

The extraction of volcanic glass shards was conducted using a stepped density separation procedure and the inert heavy liquid sodium polytungstate (SPT) (Na_6 ($H_2W_{12}O_{40}$) H_2O) (see Blockley et al., 2005). An initial 'cleaning' phase was conducted using the SPT at a specific gravity of 2.00 g/cm^3, and an 'extraction' phase using SPT with a specific gravity of 2.55 g/cm^3 (51). Material from the extraction phase was pipetted onto glass microscope slides and mounted under a coverslip using Canada balsam.

Cryptotephra identification & quantification

Microscope slides were examined using an Olympus CX-41 transmitted light microscope. Slides were traversed systematically, and counts were conducted at 20x magnification, an objective with 40x magnification was used to examine morphological detail and to assist in distinguishing volcanic glass shards from non-glass detrital 'mimics'.

No tephra was found in Column 1 or 2A, however, glass shard abundance was exceptionally high in Column 2 and in particular the scan sample T19-0206 (S6 Table 5). In order to ascertain the precise interval of greatest glass shard concentration, the four 5 cm sediment samples that comprise T19-0206 were re-sampled and processed following the methodology outlined above. To facilitate easier assay of glass shard concentrations the processed samples were spiked with *Lycopodium* spores prior to mounting onto glass slides (52). Quantification of glass shard concentrations used the following:

$$c = \left(l \times \frac{a}{b}\right) d$$

where c = concentration of glass shards, l = total *Lycopodium*, a = glass shard count, b = *Lycopodium* count, d = sample dry weight in grams.

Concentrations of glass shards were high in the four samples, however, a distinct peak in concentration was identified at a depth of 75-70 cm (S6 Table 5).

S6 Table5. Glass shard concentrations from Nahal Aqev. Whilst all intervals examined contained some glass shards, a distinct peak in concentration indicative of primary airfall, was identified at a depth of 75-70 cm.

Sample #	db Z (cm)	Unit	Dry Sample Weight (g)	Estimated glass shard counts	Notes	Dry weight (g)	Total lycopodium	Total shard count	Lycopodium spore count	Shard count per (g)
38	30-25	10	**5.473**	10's	Shards present but in lower concentrations than lower intervals					
37	35-30	10								
36	40-35	11								
35	45-40	11	**5.622**	100's	Abundance of glass shards - estimated to be less than below					
34	50-45	11								
33	55-50	11								
32	60-55	11								
31	65-60	11	**5.189**	1000+	Abundance of large platey glass shards. Some vesicular specimens also	2.604	19855	74	362	10569
30	70-65	11				2.643	19855	111	357	16316
29	75-70	11				2.627	19855	113	275	21433
28	80-75	11				2.556	19855	34	502	3437

Cryptotephra chemical characterization

The 75-70 cm interval was prepared for major element chemical characterisation using the method outlined above, but with the notable omission of the combustion stage to avoid thermal alteration of the glass shards. Following density separation, residual material was kept in de-ionised water and pipetted onto glass well-slides. Individual glass shards were extracted using a 5 µl gas chromatography syringe fitted with a 100 µm-diameter needle. These were transferred to a flat silicon sheet and impregnated in an epoxy resin to form a ‘stub’. Once hardened, the resin at the surface of the stub was removed using a series of graded silicon papers until the glass shards were exposed and sectioned. A final polish using 0.3 µm aluminium oxide powder provided a flat surface for analysis (53). The resin stub was carbon coated and analysed for major and minor elements at the WDS-EPMA (Cameca SX-100) facility at the University of Edinburgh. Probe conditions followed those of Hayward (54). Samples were analysed using a beam diameter of 8 µm set at 15 keV, and a current of 0.5 nA for Na and Al, 2 nA for Si, K, Ca, Fe and 80 nA for Mg, P, Ti, Mn. 26 successful (SiO_2 wt. % > 93%) analyses were obtained. Calibration, precision and drift was assessed by the analysis of internal Lipari and BCR-2G secondary standards.

Cryptotephra results & interpretation

The occurrence of cryptotephra within the Nahal Aqev site is exclusive to Column 2 where it forms a distinct peak within the base of Unit 11. Refinement of the ‘scan’ of cryptotephra investigation phase shows a discrete peak in glass shard concentrations in the 75-70 cm interval (S6 Table 5). Cryptotephra concentrations are of a magnitude less in the underlying interval and reduce gradually in the overlying intervals. This pattern of distribution indicates a point of primary tephra deposition within the 75-70 cm interval which can be interpreted as the result of airfall from a passing volcanic ash cloud (c.f. 55). Major and minor element chemical characterisation of the volcanic glass shards (S6 Table 6) reveals a homogenous calc-alkaline rhyolitic composition (Fig. S6.1). Chemical comparison to neighboring volcanic centres and sedimentary archives containing cryptotephras suggests the correlation to a widespread early MIS 5e tephra found in Eastern Mediterranean marine cores (see main text). Within the broad timeframe provided by the OSL dating there is a clear chemical correlation to tephra from early in the Saporpel S5 deposits of cores LC-21 and ODP-967. As shown in figure S6.2 and also summary plots in the main text this is a tight chemical match. Moreover, the chemistry of these tephra is relatively rare in the Eastern Mediterranean and is the only reported primary airfall of these highly evolved, low CaO and FeO tephra for tens of thousands of years.

S6 Table 6. Raw and normalised major and minor element glass-shard and glass standard analyses from Nahal Aqev, displayed as wt.% oxides.

Stubcode	Lab code	Analysis date	Data	SiO2 wt.%	TiO2 wt.%	Al2O3 wt.%	FeO wt.%	MnO wt.%	MgO wt.%	CaO wt.%	Na2O wt.%	K2O wt.%	P2O5 wt.%	Total wt.%
RH0976	T19-1058	24.08.20	Raw	73.691	0.067	11.137	0.378	0.056	0.055	0.566	3.825	4.207	0.020	94.000
RH0976	T19-1058	24.08.20	Raw	73.684	0.074	11.973	0.512	0.055	0.054	0.566	3.468	4.162	0.009	94.556
RH0976	T19-1058	24.08.20	Raw	74.530	0.080	11.866	0.475	0.058	0.057	0.527	3.723	3.992	0.067	95.376
RH0976	T19-1058	24.08.20	Raw	73.670	0.076	10.973	0.465	0.046	0.055	0.527	4.645	2.128	0.007	92.591
RH0976	T19-1058	24.08.20	Raw	74.252	0.069	11.474	0.532	0.061	0.054	0.574	3.743	4.121	0.009	94.890
RH0976	T19-1058	24.08.20	Raw	74.067	0.077	11.371	0.424	0.055	0.056	0.487	3.449	4.193	0.014	94.193
RH0976	T19-1058	24.08.20	Raw	74.049	0.080	11.915	0.427	0.052	0.059	0.597	3.596	4.307	0.007	95.089
RH0976	T19-1058	24.08.20	Raw	74.352	0.070	12.200	0.530	0.061	0.055	0.525	4.106	3.355	0.014	95.268
RH0976	T19-1058	24.08.20	Raw	74.004	0.068	11.412	0.471	0.060	0.056	0.557	3.899	4.009	0.007	94.542
RH0976	T19-1058	24.08.20	Raw	74.162	0.073	11.725	0.455	0.050	0.056	0.571	3.534	4.499	0.016	95.142
RH0976	T19-1058	24.08.20	Raw	73.721	0.074	11.584	0.369	0.057	0.053	0.590	3.803	4.113	0.007	94.370
RH0976	T19-1058	24.08.20	Raw	73.694	0.066	11.399	0.496	0.045	0.055	0.522	4.822	2.072	0.015	93.186
RH0976	T19-1058	24.08.20	Raw	74.540	0.073	12.211	0.557	0.067	0.053	0.526	3.794	4.099	0.011	95.930
RH0976	T19-1058	24.08.20	Raw	74.244	0.071	11.639	0.508	0.057	0.060	0.572	4.263	4.024	0.015	95.453
RH0976	T19-1058	24.08.20	Raw	74.722	0.077	11.716	0.546	0.061	0.059	0.502	3.966	3.828	0.012	95.487
RH0976	T19-1058	24.08.20	Raw	74.163	0.075	11.525	0.540	0.052	0.059	0.560	3.594	4.173	0.013	94.754
RH0976	T19-1058	24.08.20	Raw	74.188	0.073	11.996	0.501	0.058	0.056	0.567	3.791	4.202	0.016	95.448
RH0976	T19-1058	24.08.20	Raw	73.352	0.075	11.283	0.462	0.057	0.056	0.618	4.335	3.327	0.015	93.579
RH0976	T19-1058	24.08.20	Raw	73.583	0.075	12.202	0.614	0.054	0.058	0.582	4.528	2.394	0.003	94.093
RH0976	T19-1058	24.08.20	Raw	73.956	0.078	11.474	0.461	0.053	0.056	0.487	4.945	2.354	0.008	93.872
RH0976	T19-1058	24.08.20	Raw	74.780	0.072	11.675	0.593	0.063	0.052	0.532	4.452	3.175	0.018	95.411
RH0976	T19-1058	24.08.20	Raw	73.565	0.066	11.943	0.461	0.065	0.053	0.622	4.484	2.932	0.007	94.197
RH0976	T19-1058	24.08.20	Raw	73.943	0.066	11.585	0.456	0.056	0.060	0.557	5.321	2.543	0.016	94.604
RH0976	T19-1058	24.08.20	Raw	74.379	0.067	11.332	0.409	0.061	0.058	0.512	3.677	4.088	0.001	94.583

RH0976	T19-1058	24.08.20	Raw	73.830	0.076	11.869	0.482	0.051	0.059	0.630	3.700	4.007	0.007	94.711
RH0976	T19-1058	24.08.20	Raw	73.909	0.075	11.459	0.378	0.063	0.057	0.589	3.996	3.774	0.002	94.301
mean				74.039	0.073	11.651	0.481	0.057	0.056	0.556	4.056	3.618	0.013	94.601
1s.d.				0.371	0.004	0.328	0.063	0.006	0.002	0.040	0.498	0.753	0.012	0.776
2s.d.				0.743	0.009	0.655	0.126	0.011	0.004	0.079	0.995	1.506	0.024	1.552
RH0976	T19-1058	24.08.20	Normalised	78.394	0.072	11.847	0.402	0.060	0.058	0.602	4.069	4.476	0.021	100.000
RH0976	T19-1058	24.08.20	Normalised	77.926	0.078	12.662	0.541	0.058	0.057	0.599	3.668	4.402	0.009	100.000
RH0976	T19-1058	24.08.20	Normalised	78.144	0.084	12.441	0.498	0.060	0.060	0.553	3.904	4.186	0.070	100.000
RH0976	T19-1058	24.08.20	Normalised	79.564	0.082	11.851	0.502	0.049	0.060	0.569	5.017	2.298	0.007	100.000
RH0976	T19-1058	24.08.20	Normalised	78.251	0.072	12.092	0.561	0.064	0.057	0.605	3.945	4.343	0.010	100.000
RH0976	T19-1058	24.08.20	Normalised	78.633	0.082	12.072	0.450	0.058	0.060	0.517	3.662	4.452	0.015	100.000
RH0976	T19-1058	24.08.20	Normalised	77.873	0.084	12.531	0.449	0.055	0.062	0.628	3.781	4.529	0.008	100.000
RH0976	T19-1058	24.08.20	Normalised	78.045	0.073	12.805	0.557	0.064	0.058	0.551	4.310	3.522	0.015	100.000
RH0976	T19-1058	24.08.20	Normalised	78.277	0.072	12.070	0.498	0.063	0.059	0.589	4.124	4.240	0.007	100.000
RH0976	T19-1058	24.08.20	Normalised	77.949	0.077	12.324	0.478	0.053	0.059	0.600	3.715	4.729	0.017	100.000
RH0976	T19-1058	24.08.20	Normalised	78.119	0.078	12.275	0.391	0.060	0.056	0.626	4.030	4.358	0.007	100.000
RH0976	T19-1058	24.08.20	Normalised	79.083	0.071	12.233	0.533	0.048	0.059	0.560	5.175	2.224	0.016	100.000
RH0976	T19-1058	24.08.20	Normalised	77.702	0.076	12.729	0.580	0.070	0.055	0.548	3.954	4.273	0.011	100.000
RH0976	T19-1058	24.08.20	Normalised	77.781	0.075	12.194	0.532	0.059	0.062	0.600	4.466	4.216	0.016	100.000
RH0976	T19-1058	24.08.20	Normalised	78.253	0.081	12.269	0.572	0.064	0.061	0.525	4.153	4.008	0.013	100.000
RH0976	T19-1058	24.08.20	Normalised	78.270	0.079	12.163	0.569	0.055	0.062	0.591	3.793	4.404	0.013	100.000
RH0976	T19-1058	24.08.20	Normalised	77.726	0.076	12.568	0.524	0.060	0.059	0.594	3.972	4.403	0.016	100.000
RH0976	T19-1058	24.08.20	Normalised	78.385	0.080	12.057	0.494	0.060	0.060	0.660	4.632	3.555	0.016	100.000
RH0976	T19-1058	24.08.20	Normalised	78.202	0.080	12.968	0.653	0.057	0.061	0.619	4.812	2.544	0.004	100.000
RH0976	T19-1058	24.08.20	Normalised	78.784	0.083	12.223	0.491	0.056	0.060	0.519	5.268	2.508	0.008	100.000
RH0976	T19-1058	24.08.20	Normalised	78.377	0.076	12.236	0.621	0.066	0.054	0.557	4.666	3.327	0.018	100.000
RH0976	T19-1058	24.08.20	Normalised	78.096	0.070	12.679	0.489	0.069	0.056	0.660	4.760	3.113	0.007	100.000

RH0976	T19-1058	24.08.20	Normalised	78.161	0.070	12.246	0.482	0.059	0.064	0.589	5.624	2.688	0.017	100.000
RH0976	T19-1058	24.08.20	Normalised	78.639	0.070	11.981	0.432	0.065	0.061	0.541	3.887	4.322	0.001	100.000
RH0976	T19-1058	24.08.20	Normalised	77.953	0.080	12.532	0.509	0.054	0.063	0.665	3.907	4.231	0.007	100.000
RH0976	T19-1058	24.08.20	Normalised	78.376	0.079	12.152	0.401	0.066	0.061	0.624	4.237	4.002	0.002	100.000
mean				78.27	0.08	12.32	0.51	0.06	0.06	0.59	4.29	3.82	0.01	100.00
1s.d.				0.42	0.00	0.29	0.07	0.01	0.00	0.04	0.54	0.78	0.01	0.00
2s.d.				0.84	0.01	0.59	0.13	0.01	0.00	0.08	1.09	1.56	0.03	0.00
Glass standards	Lipari	24.08.20		74.629	0.080	13.356	1.459	0.068	0.061	0.770	4.079	5.267	0.013	99.783
Glass standards	Lipari	24.08.20		74.816	0.080	12.992	1.575	0.069	0.034	0.780	4.103	5.093	0.013	99.555
Glass standards	Lipari	24.08.20		74.535	0.078	13.109	1.611	0.075	0.034	0.753	4.191	5.126	0.024	99.534
mean				74.66	0.08	13.15	1.55	0.07	0.04	0.77	4.12	5.16	0.02	99.62
1s.d.				0.14	0.00	0.19	0.08	0.00	0.02	0.01	0.06	0.09	0.01	0.14
2s.d.				0.29	0.00	0.37	0.16	0.01	0.03	0.03	0.12	0.19	0.01	0.28
Glass standards	BCR2g	24.08.20		54.746	2.229	13.864	12.443	0.183	3.718	7.360	3.275	1.889	0.340	100.048
Glass standards	BCR2g	24.08.20		54.308	2.220	13.848	12.216	0.198	3.742	7.158	3.250	1.839	0.350	99.130
Glass standards	BCR2g	24.08.20		55.536	2.215	13.323	12.783	0.197	3.608	7.451	3.147	1.797	0.345	100.403
mean				54.86	2.22	13.68	12.48	0.19	3.69	7.32	3.22	1.84	0.35	99.86
1s.d.				0.62	0.01	0.31	0.29	0.01	0.07	0.15	0.07	0.05	0.00	0.66
2s.d.				1.24	0.01	0.62	0.57	0.02	0.14	0.30	0.14	0.09	0.01	1.31

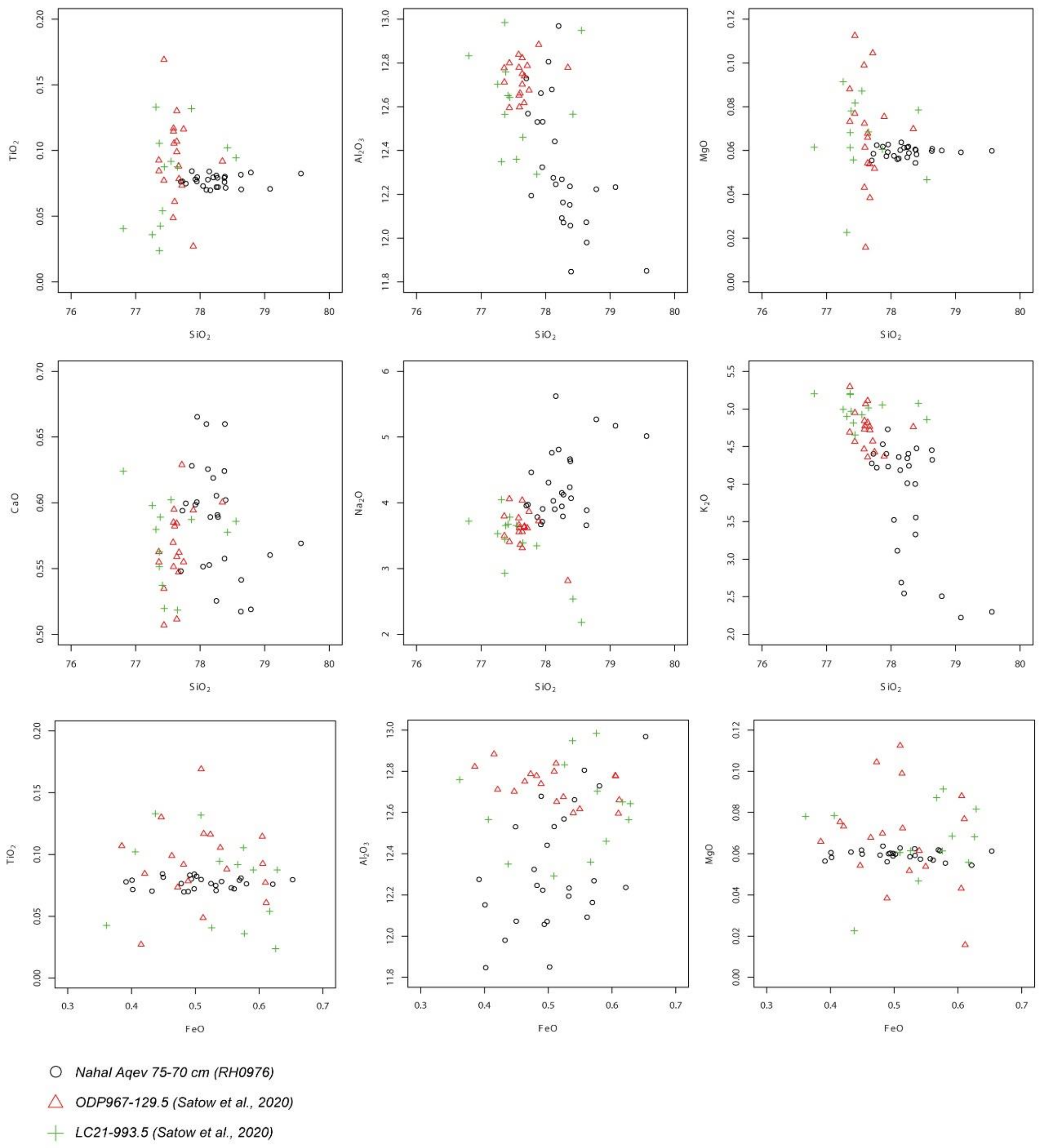

Figure S6.2. Chemical bi-plots of major and minor element chemistry from Nahal Aqev, LC-21 and ODP-967 tephra (values normalized on water free basis).

References

1. Greenbaum, N. Schick, A. P. & Baker V. R., The palaeoflood record of a hyperarid catchment, Nahal Zin, Negev Desert, Israel. *Earth Surface Processes and Landforms* 25(9), 951-971 (2000).
2. Avni, Y., Tectonic setting and physiography setting of the Levant in *Quaternary of the Levant* (eds. Enzel Y. & Bar-Yosef O.) 3-16 (Cambridge University Press, 2017).
3. Avni, Y. & Zilberman E., Landscape evolution triggered by neotectonics in the Sede Zin region, central Negev, Israel. *Isr. J. Earth Sci.* 55, 189-208 (2007).
4. Avni, Y. Bartov, Y. Garfunkel, Z. & Ginat H. The evolution of the Paran drainage basin and its relation to the Plio-Pleistocene history of the Arava Rift western margin. Israel. *Isr. J. Earth Sci.* 49, 215-238 (2000).
5. Avni, Y. & Wieler N., *The Geological Map of Israel 1:50,000. Sede Boqer Sheet*. (Geological Survey of Israel, 2013).
6. Avni, Y. Faershtein, G. & Porat, N. Studies of stream terraces in the Negev Highlands and their relationship with the Levant alluvial chronologist in Quaternary of the Levant (eds. Enzel Y. & Bar-Yosef O.) 457–469 (Cambridge University Press, 2017).
7. Avni, Y. Oron, M. Cohen-Sasson, E. Porat, N. & Barzilai, O. Chrono-sequences of alluvial terraces and fossilized water bodies as a predictive model for detecting Lower and Middle Palaeolithic sites in the Negev desert, Israel. Quat. Sci. Rev. 268 https://doi.org/10.1016/j.quascirev.2021.107114 (2021).
8. Crouvi O. et al., Significance of primary hilltop loess in reconstructing dust chronology, accretion rates and sources: an example from the Negev desert, Israel. *J. Geophys. Res.* 114 (F02017), 1-16 (2009).
9. Avni, Y. Porat, N. Plakht, J. & Avni, G. Geomorphologic changes leading to natural desertification processes versus anthropogenic land conservation in an arid environment, the Negev Highlands, Israel. *Geomorphology* 82, 177-200 (2006).
10. Faershtein, G. Porat, N. Avni, Y. & Matmon, A. Aggradation-incision transition in arid environments at the end of the Pleistocene: an example from the Negev Highlands, southern Israel. *Geomorphology* 253, 289-304 (2016).
11. Schwarcz, H. P. Blackwell, B. Goldberg, P. & Marks, A. E. Uranium series dating of travertine from archaeological sites, Nahal Zin, Israel. Nature 277(5697), 558–560 (1979).

12. Marks, A. E. *Prehistory and Paleoenvironments in the Central Negev, Israel, Volume I: The Avdat/Aqev Area, Part 1* (SMU Press 1976)
13. Marks, A. E. *Prehistory and Paleoenvironments in the Central Negev, Israel: The Avdat/Aqev Area, Part 2 and the Har Harif* (SMU Press, 1977).
14. Marks, A. E. *Prehistory and Paleoenvironments in the Central Negev, Israel, Volume III: The Avdat/Aqev Area, Part 3*. (SMU Press, 1983).
15. Crew, H. L. The Mousterian site of Rosh Ein Mor in *Prehistory and Paleoenvironments of the Central Negev, Israel*, *Vol. I, The Avdat/Aqev Area, Part 1*, (ed. A. E. Marks) 75–112 (SMU Press, 1976).
16. Munday, F. C. Intersite variability in the Mousterian occupation of the Avdat/Aqev area in *Prehistory and Paleoenvironments of the Central Negev, Israel, Vol. I,* (ed. A. E. Marks) 57–68 (SMU Press 1976).
17. Munday, F. C. Nahal Aqev (D35): a stratified, open-air Mousterian occupation in the Avdat/Aqev area in *Prehistory and Paleoenvironments of the Central Negev, Israel, Vol. II*, (ed. A. E. Marks) 35–60 (SMU Press, 1977).
18. Marks, A. E. & Freidel, D. A. Prehistoric settlement patterns in the Avdat/Aqev area in *Prehistory and paleoenvironments in the Central Negev, Israel, Vol II* (ed. A. E. Marks) 131-158 (SMU Press 1977).
19. Marks, A. E. & Rose, J. I. A century of research into the origins of the Upper Paleolithic in the Levant in *Néandertal/Cro-Magnon. La Rencontre* (ed. M. Otte) 221–266 (Errance, Arles 2014).
20. Rink W. J. et al., Age of the Middle Palaeolithic site of Rosh Ein Mor, Central Negev, Israel: Implications for the age range of the early Levantine Mousterian of the Levantine corridor. *J. Archaeol. Sci.* 30(2), 195–204 (2003).
21. Richter, D. Advantages and limitations of thermoluminescence dating of heated flint from Paleolithic sites*. Geoarchaeology* 22(6), 671–683 (2007).
22. Goder-Goldberger, M. & Bar-Matthews, M. Novel chrono-cultural constraints for the Middle Paleolithic site of Rosh Ein Mor (D15), Israel*. J. Archaeol. Sci. Rep.*, 24, 102-114. (2019).
23. Barzilai, O. & Boaretto, E. Nahal 'Aqev. *Hadashot Arkheologiot 133* https://www.hadashot-esi.org.il/Report_Detail_Eng.aspx?id=25928 (Accessed 18 September 2021).

24. Goldberg, P. Nahal Aqev (D35) stratigraphy and environment of deposition (appendix) in *Prehistory and Paleoenvironments of the Central Negev, Israel, Vol. II*, (ed. A. E. Marks) 56–60 (SMU Press, 1977).
25. Tsartsidou G. et al., The phytolith archaeological record: Strengths and weaknesses evaluated based on a quantitative modern reference collection from Greece. *J. Archaeol. Sci.* 34, 1262–1275 (2007).
26. Weiner, S. *Microarchaeology: Beyond the Visible Archaeological Record* (Cambridge University Press, 2010).
27. Weiner, S. Pinkas, I. Kossoy, A. & Feldman, Y. Calcium sulfate hemihydrate (bassanite) crystals in the wood of the Tamarix tree. *Minerals* 11, 289-297 (2021).
28. Shahack-Gross, R. & Finkelstein, I. Subsistence practices in an arid environment: a geoarchaeological investigation in an Iron Age site, the Negev Highlands, Israel. *J. Archaeol. Sci.* 35(4), 965-982 (2008).
29. F. Berna, et al., Sediments exposed to high temperatures: reconstructing pyrotechnological processes in Late Bronze and Iron Age Strata at Tel Dor (Israel). *J. Archaeol. Sci.* 34, 358-373 (2007).
30. Benjamini, C. Planktonic foraminiferal biostratigraphy of the'Avedat Group (Eocene) in the northern Negev, Israel *J. Paleontol.* 325-358 (1980).
31. Lucke B. et al., Composition of Holocene dust and aeolian sediments in archaeological structures of the southern Levant. *Atmosphere* 10, 762 https://doi.org/10.3390/atmos10120762 (2019).
32. Crouvi O. et al., Significance of primary hilltop loess in reconstructing dust chronology, accretion rates, and sources: An example from the Negev Desert, Israel. *J. Geophys. Res.* 114: F02017 (2009).
33. Porat, N. Use of magnetic separation for purifying quartz for luminescence dating. *Ancient TL* 24, 33-36(2006).
34. Porat, N. Faerstein, G. Medialdea, A. & Murray, A.S. Re-examination of common extraction and purification methods of quartz and feldspar for luminescence dating. *Ancient TL* 33, 22-30. (2015).
35. Murray, A.S. & Wintle, A.G. Luminescence dating of quartz using an improved single aliquot regenerative-dose protocol. *Radiat. Meas* 32, 57-73 (2000).

36. Hansen, V. A. et al. A new irradiated quartz for beta source calibration. *Radiat. Meas* 81, 123-127 (2015).
37. Porat, N. et al. OSL Dating in Multi-Strata Tel: Megiddo (Israel) as a Case Study. Quat. Geochronol 10, 359-366. (2012).
38. Porat, N. Rosen, S.A. Avni, Y. Boaretto, E. Dating the Ramat Saharonim Late Neolithic Desert Cult site. *J. Archaeol. Sci*. 33, 1341-1355 (2006).
39. Thiel, C. et al., Luminescence dating of the Stratzing loess profile (Austria) - testing the potential of an elevated temperature post-IR IRSL protocol. Quat. Int. 234(1-2), 23-31 (2011).
40. Faershtein, G. Porat, N. & Matmon, A. Extended-range luminescence dating of quartz and alkali feldspar from aeolian sediments in the eastern Mediterranean. *Geochronology* 2, 1–18; https://doi.org/10.5194/gchron-2-1-2020 (2020).
41. Buylaert, J. P. Murray, A. S. & Huot, S. Optical dating of an Eemian site in Northern Russia using K-feldspar. *Radiat. Meas* 43, 715-720 (2008).
42. Huntley, D. J. & Lamothe, M. Ubiquity of anomalous fading in K-feldspars and the measurement and correction for it in optical dating, *Canadian J. Earth Sci.* 38, 1093–1106 (2021).
43. Galbraith, R. F. & Roberts, R. G. Statistical aspects of equivalent dose and error calculation and display in OSL dating: an overview and some recommendations. *Quat. Geochronol* 11, 1-27 (2012).
44. Balescu, S. et al. Luminescence dating of a gigantic palaeolandslide in the Gobi-Altay mountains, Mongolia*, Quat. Geochronol* 2, 290-295 (2007).
45. Rendell, H. Yair A. &Tsoar H. Thermoluminescence dating of period of sand movement and linear dune formation in the northern Negev, Israel in *The Dynamics and Environmental Context of Aeolian Sedimentary Systems* (ed.K. Pye) 69-74 (1993).
46. Nambi, K. S. V. & Aitken, M. J. Annual dose conversion factors for TL and ESR dating. *Archaeometry* 28: 202–205 (1986).
47. Wintle, A. G. & Murray, A. S. A review of quartz optically stimulated luminescence characteristics and their relevance in single aliquot regeneration dating protocols. *Radiation Measurements* 41: 369–391 (2006).
48. Faershtein, G. Porat, N. & Matmon, A. Natural saturation of OSL and TT-OSL signals of quartz grains from Nilotic origin. *Quat. Geochronol* 49, 146-152 (2019).

49. Duller, G. A. T. Single-grain optical dating of Quaternary sediments: why aliquot size matters in luminescence dating. *Boreas*, 37, 589–612 (2008).
50. Buylaert J. P. et al., A robust feldspar luminescence dating method for Middle and Late Pleistocene sediments. *Boreas,* 41, 435–451. (2012).
51. Blockley S. P. E. et al., A new and less destructive laboratory procedure for the physical separation of distal glass tephra shards from sediments. *Quat. Sci. Rev*. 24, 1952–1960 (2005).
52. Gehrels, M. J. et al., Towards rapid assay of cryptotephra in peat cores: review and evaluation of various methods. *Quat. Int.* 178, 68-84 (2008).
53. Hall, M. & Hayward, C. Preparation of micro-and crypto-tephras for quantitative microbeam analysis. *Geol. Soc. Spec. Publ.* 398, 21-28 (2014).
54. Hayward, C. High spatial resolution electron probe microanalysis of tephras and melt inclusions without beam-induced chemical modification. The Holocene 22, 119–125 (2012).
55. Davies, S. M. Cryptotephras: the revolution in correlation and precision dating. *Journal of Quaternary Science* 30, 114-130 (2015).
